# Mitochondrial Lon-induced mitophagy benefits hypoxic resistance via Ca^2+^-dependent FUNDC1 phosphorylation at the ER-mitochondria interface

**DOI:** 10.1038/s41419-023-05723-1

**Published:** 2023-03-16

**Authors:** Ananth Ponneri Babuharisankar, Cheng-Liang Kuo, Han-Yu Chou, Vidhya Tangeda, Chi-Chen Fan, Chung-Hsing Chen, Yung-Hsi Kao, Alan Yueh-Luen Lee

**Affiliations:** 1 PhD program in molecular medicine, NHRI & NCU, Taoyuan, Taiwan; 2 National Institute of Cancer Research, National Health Research Institutes, Zhunan, Miaoli, 35053 Taiwan; 3 Department of Life Sciences, College of Health Sciences & Technology, National Central University, Zhongli, Taoyuan, 32001 Taiwan; 4 Department of Medical Laboratory Science and Biotechnology, Yuanpei University of Medical Technology, Hsinchu, 300 Taiwan; 5 Graduate Institute of Biomedical Sciences, China Medical University, Taichung, 40402 Taiwan; 6 Department of Biotechnology, College of Life Science, Kaohsiung Medical University, Kaohsiung, 80708 Taiwan

**Keywords:** Mitochondria, Mitophagy

## Abstract

During hypoxia, FUNDC1 acts as a mitophagy receptor and accumulates at the ER (endoplasmic reticulum)-mitochondria contact sites (EMC), also called mitochondria-associated membranes (MAM). In mitophagy, the ULK1 complex phosphorylates FUNDC1(S17) at the EMC site. However, how mitochondria sense the stress and send the signal from the inside to the outside of mitochondria to trigger mitophagy is still unclear. Mitochondrial Lon was reported to be localized at the EMC under stress although the function remained unknown. In this study, we explored the mechanism of how mitochondrial sensors of hypoxia trigger and stabilize the FUNDC1-ULK1 complex by Lon in the EMC for cell survival and cancer progression. We demonstrated that Lon is accumulated in the EMC and associated with FUNDC1-ULK1 complex to induce mitophagy via chaperone activity under hypoxia. Intriguingly, we found that Lon-induced mitophagy is through binding with mitochondrial Na^+^/Ca^2+^ exchanger (NCLX) to promote FUNDC1-ULK1-mediated mitophagy at the EMC site in vitro and in vivo. Accordingly, our findings highlight a novel mechanism responsible for mitophagy initiation under hypoxia by chaperone Lon in mitochondria through the interaction with FUNDC1-ULK1 complex at the EMC site. These findings provide a direct correlation between Lon and mitophagy on cell survival and cancer progression.

## Introduction

Autophagy, a critical degradation mechanism of various intracellular components through the formation of double-bilayered membrane vesicles called autophagosomes and its fusion with lysosomes is necessary for maintaining the cellular homeostasis. Macroautophagy, a kind of autophagy, is important for its selective degradation and vital for the cell survival function through autophagosomes formation around specific cellular organelles or molecular components, including mitochondria, endoplasmic reticulum (ER), Peroxisome, Golgi and pathogen [1, 2]. Mitochondrial autophagy (mitophagy) removes damaged and surplus mitochondria, which plays a vital role in mitochondrial quality control. Mitochondria are the essential components for energy metabolic pathways and cell survival; they are dynamically regulated by the mitochondrial fission and fusion. Thus, quality control of mitochondria is important for cell survival under both physiological and stress/pathological conditions like starvation, oxidative stress, and hypoxia, whereas its dysfunction was reported to cause several serious diseases like neurodegeneration, diabetes, cardiovascular disorders, and cancer [3–9].

The initiation of autophagy/mitophagy is mediated by the ULK1 (Unc-51 like kinase), a serine/threonine-specific protein kinase, and its associated protein complex with HORMA domain-containing autophagy-related protein 13 (ATG13), scaffold FIP200 (RB1CC1), and autophagy-related protein 101 (ATG101) in mammalian cells [10, 11]. The upstream regulation of ULK1 by AMPK/mTOR has been extensively studied indicating ULK1 significance on deciding the on/off autophagy status. Phosphorylation of ULK1 complex and subsequent phosphorylation of Vps34-ATG14-Beclin1 complex by ULK1 are important for the phagophore nucleation formation through the ER cradle associated omegasomes [12–15]. In response to hypoxia, FUNDC1, a mitochondrial outer membrane protein, acts as a mitochondrial receptor to mediate hypoxia-induced mitophagy [16]. FUNDC1 accumulates at the ER-mitochondria contact sites (EMC), also called mitochondria-associated membranes (MAM), in response to hypoxia, recruiting Drp1 to ensure hypoxia-induced mitochondrial fission [17] and subsequently interacting with LC3 to complete mitophagy. The ER-mitochondria contact sites have been demonstrated to be involved in autophagy, Ca^2+^ transport, and lipid metabolism, signifying the fact that organelle communication is important for the cellular bioenergetics. The control of cytosolic Ca^2+^ oscillations by NCLX was associated with many pathological conditions including cancer. The sustained intracellular Ca^2+^ levels in cancer cells can activate the AMPK-ULK1 phosphorylation and FUNDC1 signaling during hypoxia for mitophagy [18, 19], highlights the significance of calcium-mitophagy coordination in regulating the cell proliferation during various stress conditions.

Mitochondrial Lon is a multi-function and stress-induced protein and where uses its protease, ATPase, DNA binding [20–22], and chaperone activities [23–25] to participate in the protein quality control and stress response pathways in mitochondria [26–28]. Lon is upregulated under hypoxia and oxidative stress [29, 30] and generates ROS to promote multifarious important cellular processes in tumorigenesis [29, 31]. Lon utilizes chaperone activity to stabilize its clients, mitochondrial p53 [23], mtHsp60-Hsp70 complex [24], PYCR1 [31], and mitochondrial Na^+^/Ca^2+^ exchanger (NCLX) [32], which is involved in the cell survival upon ROS stress. Intriguingly, a recent paper found that the translocation of Lon to the EMC was observed when cells was treated with Efavirinez, a non-nucleoside reverse transcriptase inhibitor [33], which causes both ER and mitochondrial stress [34]. However, the functional significance of Lon in the EMC and where how Lon promotes hypoxia-induced mitophagy has been remained poorly understood.

The present study aimed at delineating the mechanism responsible for the role of mitochondrial Lon in the EMC and in the initiation of mitophagy under hypoxia. Here, we report that chaperone Lon physically interacts with NCLX and FUNDC1-ULK1 complex. These interactions further enhance the ULK1 stability and its kinase activity to phosphorylate its interacting partners ATG13 (Serine 355), Beclin1 (Serine 15), and FUNDC1 (Serine 17) respectively. Accordingly, all these events responsible for mitophagy initiation are elucidated by the translocation of Lon from the matrix to the EMC region upon hypoxia stress, which provides the triggering mechanism from inside mitochondria to outside contacts. These findings define an important function of chaperone Lon that integrates calcium-mitophagy coordination, and it governs the function of mitochondrial Lon in cell survival and drug resistance upon hypoxia-induced mitophagy providing a future choice of cancer therapy for patients.

## Material and methods

### Cell culture and cell treatment

HCT-15 colon cancer cells were cultured in medium containing Roswell Park Memorial Institute (RPMI) (GIBCO, New York, NY, USA) and oral cancer cells like OEC-M1 and HSC-3, cells were cultured in medium containing Dulbecco’s modified Eagle’s essential medium (DMEM) (GIBCO, New York, NY, USA), supplemented with 10% Fetal Bovine Serum (FBS) (FBS qualified; Invitrogen) and penicillin/streptomycin (50 U/mL; sigma, St. Louis, MO, USA) in a 37 °C humidified incubator with 5% CO_2_.

### Reagents and Antibodies

Antibodies to human Lon were produced as described previously [35]. The following primary antibodies were used in this study: Autophagy Induction Antibody Sampler Kit containing anti-ULK1 monoclonal antibody, anti-p-ULK1 S555, anti-ATG13, anti-p-ATG13 S355, anti-FIP200, anti-ATG101. Anti-NFkB anti-LC3B, anti-LAMP1, anti-VDAC, anti-Calnexin, anti-Tubulin, and ULK1 antibody sampler Kit containing anti-ATG14, anti-p-ATG14 S29, anti-Beclin1, anti-p-Beclin1 S15, were purchased from Cell signaling technology. Anti-FACL4, anti-GAPDH, and anti-β-actin were purchased from Gentex also the Gentex helped in producing in-house antibody anti-FUNDC1 and anti-p-FUNDC1 S17 which were raised in rabbit and purified. Anti-HSP60 and anti-Aconitase2 from santa cruz. Anti-myc and and anti-Flag were obtained from Merck Millipore. Anti-HIF1α was obtained from Life science bio. Anti-ULK1 for immuofluorescence studies was obtained from Santa cruz. Cobalt chloride (CoCl_2_), SBI-0206965, Bafilomycin A1 and N-Acetyl Cysteine (NAC) were purchased from sigma, dissolved in DMSO and stored at −20 °C.

### Patients and clinical sample

Tissue specimens of 92 patients with oral squamous cell carcinoma (OSCC) were used for immunohistochemistry (IHC) analysis based on the availability of archival human tissue blocks from diagnostic resection specimens in the Departments of Pathology at Mackay Memorial Hospital, Taipei, Taiwan with approval from the Institutional Review Board (IRB numbers 15MMHIS046 and 17MMHIS085). The main clinical characteristics of the 92 patients selected for this study are shown in a previous study [23]. All experiments were performed in accordance with relevant guidelines and regulations.

### Western blotting

The cells were harvested by trypsinization and lysed with NETN buffer (20 mM Tris (pH 8.0), 1 mM EDTA, 150 mM NaCl, 0.5% nonidet P-40 (NP-40)) containing protease inhibitor cocktail (Roche, Mannheim, Germany). The cell lysates were then centrifuged at 10,000×*g* at 4 °C to obtain solubilized cellular proteins. Protein was quantified with a bicinchoninic acid protein assay (Pierce, Rockford, IL, USA) according to the manufacturer’s instructions. Proteins were separated by 8% or 10% or 12% SDS-PAGE and electro-transfered to a polyvinylidene fluoride membrane. Target proteins were detected by incubating with the indicated primary antibodies, followed by the corresponding HRP-conjugated secondary antibodies. Immunoreactive bands were detected with Immobilon Western Chemiluminescent HRP Substrate (Millipore).

### Immunoprecipitation

HCT-15 cells seeded in 10 cm dish were co-transfected with specific constructs to overexpress proteins of interest for 24–48 h. NETN (150 mM NaCl, 1 mM EDTA, 20 mM Tris-Cl (pH 8.0), 0.5% NP-40) lysis buffer containing protease and phosphatase inhibitors (1.0 mM sodium orthovanadate, 50 μM sodium fluoride). Samples were incubated on ice with intermittent agitation by pipetting for 30 min. Beads were equilibrated using bead resuspension buffer (150 mM Tris-buffered Saline, 50 mM NaCl, 0.25% Tween). Protein lysates were precleared by centrifugation at 4 °C for 10 min at 15,000×*g*. Clarified lysates were incubated with specific equilibrated beads for various periods at 4 °C. Beads were then washed with ice-cold Wash buffer (150 mM Tris-buffered Saline, 50 mM NaCl) 3–5 times. Clarified lysates were first incubated with antibodies specified in figure legends for 24 h at 4 °C. A/G beads (Pierce) were equilibrated with resuspension buffer then incubated with antibody-lysate mix for 1 h at 4 °C. Beads was washed as above. Bound samples were eluted using 4X sample buffer. Samples were processed for immunoblotting for examining the binding partners.

### Subcellular fractionation

Previously the detailed protocol for the EMC/MAM fractionation has been described (Wieckowski et al.; [36]). In brief, 10 number of HCT-15 confluent plates (about 15-cm diameter) were collected and resuspended in ice-cold IB cells-1 buffer containing 225 mM mannitol, 75 mM sucrose, 0.1 mM EGTA, and 30 mM Tris–HCl pH 7.4. After, gentle and slow homogenization of resuspended cells by slow stokes about 200 times using the dounce homogenizer and homogenized extracts were subjected for centrifugation at 600×*g* for 5 min at 4 °C. Repeat the centrifugation with the collected supernatant until no traces of pellet is seen (PNS). The collected supernatants were transferred to a multiple 1.5 mL centrifuge tubes and centrifuge at 7000×*g* for 10 min at 4 °C to separate the crude mitochondrial pellet and supernatant containing cytosol and ER. Repeat the centrifugation with the collected supernatant until no traces of pellet to avoid the EMC/MAM and mitochondrial contamination. Further ultracentrifugation of the collected supernatant at 100,000×*g* for 1 h isolates the ER (pellet) and cytosol (supernatant). The crude mitochondrial pellet was resuspended gently in 2 ml of ice-cold mitochondrial resuspension buffer (MRB) containing 250 mM mannitol, 5 mM HEPES pH 7.4, and 0.5 mM EGTA which added on top of 8 ml of Percoll medium in an ultracentrifuge tube. MRB solution was then layered gently on top of the mitochondrial suspension to fill the centrifuge tube. At final step, centrifugation was carried out at 95,000×*g* for 60 min at 4 °C to isolate the EMC/MAM and pure mitochondria and to obtain pure EMC/MAM the collected supernatant was further ultracentrifuged at 100,000×*g* for 1 h.

### Immunofluorescence microscopy

Cells seeded in 6-well plate (corning) were treated as indicated in figure legends. After treatment, cells were rinsed in PBS and fixed with 4% paraformaldehyde at RT for 10 min. Cells were washed with PBS. For immunostaining, cells were then permeabilized with methanol for 10 min, and blocked with 1% BSA in TBS or PBS for 1 h min at RT. After incubation, cells were supplemented with antibodies (1:1000) overnight at 4 °C. Cells were then washed with PBS 3 times and incubated with Alexa 488 or 546, 405-conjugated anti-mouse or anti-rabbit secondary antibodies (Invitrogen). Finally, coverslips were mounted by Pro-Long® Gold Antifade Reagent with DAPI (Invitrogen, Carlsbad, CA) for room temperature 10 min. Fluorescent Cell images were captured with Olympus DP70 upright microscope. LC3B counting was done using cell counter macros for FIJI software (semi-automatic quantification).

For confocal imaging of mitophagy assay using mito-QC, HCT-15 cells were transiently transfected with mito-QC to express fluorescent tagged mcherry and GFP proteins under CoCl_2_ or Lon expressed cells. After treatments, cells were fixed, permeabilized, blocked and treated with corresponding primary and secondary antibodies as above and washed 3 times with PBS prior to image analysis. The cell treatment with anti-LAMP1 antibody to study the overlapping with mCherry signals. Also, the Confocal imaging were performed for the method indicated in figure legends (Fig. 5C–E). Images were taken using a ×63 oil objective on an SP5II Leica confocal microscope and the images were processed using LASX software/canvasX16 and Imaris (version 9.5 -Bit plane mode -3D surface construction) software. Images were digitally altered within linear parameters, with minimal adjustments to levels and linear contrast applied to all images.

### Transmission electron microscopy

The experiments were performed as previously described by reported protocols. Cells after respective antibodies treatment were fixed in 2% glutaraldehyde in 0.1 M phosphate buffer, and a 2% phosphotungstic acid solution (pH 7.0) was used for negative staining. Negative staining was used for the single-droplet negative staining technique on continuous and holey carbon support films. All transmission electron microscopy (TEM) procedures were performed by Bio Materials Analysis Technology (Bio MA-Tek, Taiwan).

### Calcium assay

Calcium assay was performed as described previously [32]. In brief, to measure cytosol or mitochondrial calcium, OEC-M1 cells were treated with CoCl_2_ (18 h) or transfected with Vector, Lon, shNCLX (48 h) in presence or absence of CGP37157 (10 µM - 4 h). After incubation, cells were washed using HBSS with Ca^2+^ and Mg^2+^ for three times and incubated with cytosolic calcium probe Fura-2 AM (1 µM) (molecular probes) for 30 mins at room temp. After incubation, cells were washed thrice with HBSS buffer and imaged under Leica microscope. Cells were imaged under 20X lens and captured using CCD camera every 0.5 s. Excitation was done at 340 and 380 nm alternatively and emission at 510 nm. Basal calcium levels were measured in presence of buffer using ATP agonizts to measure calcium. Analysis was done using LASX software and data was expressed as the ratio of 380/340 subtracted from background ROI. Cells were imaged under 40 × 1.5 numerical aperture and a CCD camera was used to capture the images every 2 sec. In case of mitochondrial calcium, cells will be transducted with a lentiviral plasmid mt-lar-GECO for 24 h and replace medium before transfection or drug treatment. (Ca^2+^) mito were measured by mt-lar-GECO with excitation 560/40 nm and emission 645/75 nm. First basal levels were measured in presence of HBSS buffer for 60 sec ATP agonist was added and made further readings. (Ca2 + ) mito changes were quantified as (F-F0)/F0 where F is fluorescence intensity at each time point and F0 is the average fluorescence intensity of basal calcium. Analysis was done using LASX software by choosing ROI. For cytosolic calcium, data expressed as the ratio of 488/380 subtracted from background ROI.

### Cell viability assay

The HCT-15 cells were seeded in 96-well plates and were treated with CoCl_2_ or cells transiently expressing Lon. After the treatment time the respective cell conditions were treated with SBI-0206965 (20 μM) or BafilomycinA1 (100 nM) for 12–24 h. Later, MTS reagent about 120 μl was added to each condition mentioned in the figure legend and incubated for 90 min. The absorbance was read at 490 nm and the results were quantified using Graphpad prism 9.0.

### Apoptosis assay

Apoptosis was analyzed by caspase 3-dependent NucView® 488 caspase 3 assay kit (#30029 - Biotium, Inc. San Francisco, USA). NucView® 488 Caspase-3 substrate is a novel cell membrane-permeable fluorogenic caspase substrate designed for detecting caspase-3 activity within live cells in real time. The cells were seeded in 12 well plates and were treated with CoCl_2_ (200 μM) and pCDNA3-myc-Lon for 18 and 36 h respectively. After incubation the cells were subjected for treatment with SBI-0206965 and BafilomycinA1 in both CoCl_2_ treated/ Lon expressed cells. Finally, the NucView® 488 Caspase-3 substrate was added to all the treatment/no treatment conditions and incubated according to manufacturer’s instructions. The apoptosis-based fluorescence image were captured using Olympus DP80 inverted microscope using 40X objective.

### Immunohistochemistry, IHC

IHC was performed as described previously [24, 29].

### Statistical analysis

For statistics, cells were randomly selected to calculate the number of MAM, gold particles, and colocalizations. Assays for characterizing cell phenotypes were analyzed by Student’s t‐test, and correlations between groups were calculated using Pearson’s test for colocalization experiments. *P*‐values < 0.01 were deemed statistically significant. All statistical data were calculated with GraphPad Prism software.

## Results

### Hypoxia induces ULK1-related autophagy and mitochondrial Lon in cancer cells

**Fig. 1:**
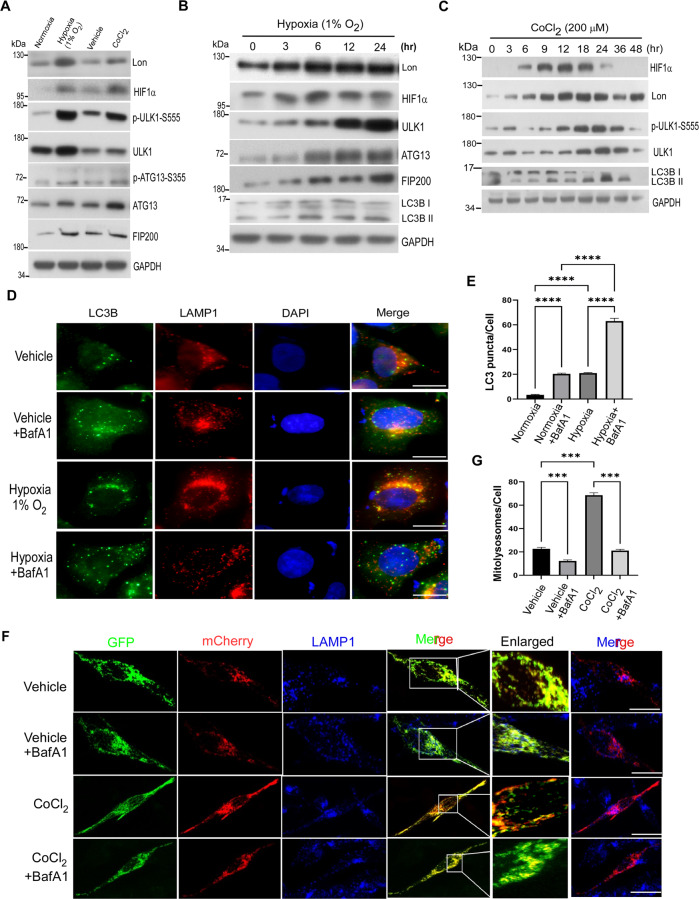
Hypoxia induces ULK1-related autophagy and mitochondrial Lon in cancer cells. **A** HCT-15 cells were exposed to hypoxia (1% O_2_) for 24 h or CoCl_2_ (200 µM) for 16 h and the collected lysates were immunoblotted against the mitophagy signaling using indicated antibodies. GAPDH as the loading control. **B** HCT-15 cells were exposed to hypoxia (1% O_2_) for the indicated time points and the protein levels of ULK1 signaling and LC3B were obtained after immunoblotting. Cell lysates were analyzed by immunoblotting using indicated antibodies. GAPDH as the loading control. **C** HCT-15 cells were treated with CoCl_2_ (200 μM) for different time points (0–48 h). Cell lysates were analyzed by immunoblotting using the indicated antibodies. GAPDH as the loading control. **D**, **E** Hypoxia mimic induces lysosome-mediated autophagy. **D** Hypoxia mimic-induced lysosome-mediated autophagy were verified by immunofluorescence. HCT-15 cells were treated with or without CoCl_2_ (200 μM for 18 h) and with BafilomycinA1 (100 nM for 6 h) or not. The cells were fixed and immunostained by LC3B (green) and anti-LAMP1 (red) antibodies. DNA was stained with DAPI (blue). Scale bars = 50 μm. **E** Hypoxia mimic-induced lysosome-mediated autophagy were quantified by LC3B puncta/cell detection according to the immunofluorescence data in D. LC3B puncta/cell was quantified by selecting more than 50 cells per condition. *n* = 3 biological replicates. The error bars shown in the panel represent the standard deviation from three independent experiments. ***p < 0.001. **F**, **G** Hypoxia mimic induces lysosome-mediated mitophagy. **F** Hypoxia mimic-induced lysosome-mediated mitophagy were verified by confocal immunofluorescence. HCT-15 cells transiently expressing mito-QC were treated with or without CoCl_2_ (200 μM for 18 h) and with BafilomycinA1 (100 nM for 6 h) or not. The cells were fixed and immunostained by GFP (mitochondria, green), mCherry (mitolysosome, red), and anti-LAMP1 (lysosome, blue) antibodies. Scale bars = 10 μm. **G** Hypoxia mimic-induced lysosome-mediated mitophagy were quantified by mcherry signals according to the immunofluorescence data in F. *n* = 3 biological replicates. The error bars shown in the panel represent the standard deviation from three independent experiments. ***p < 0.001.

During hypoxia-induced mitophagy, FUNDC1-ULK1 plays a critical role in regulating other autophagy-related proteins to mediate mitophagy [37, 38]. To explore the mechanism responsible for Lon in the initiation of mitophagy under hypoxia, the level of hypoxia-inducible factor-1α (HIF-1α), Lon, ULK1, and ULK1 downstream autophagy proteins was examined under physiological hypoxia condition. As expected [30], the level of HIF-1α, Lon, ULK1, and ULK1 downstream autophagy proteins, ULK1-S555 phosphorylation, ATG13, and FIP200, was increased under hypoxia exposure or CoCl_2_ treatment in HCT-15 colorectal cancer cells and in FADU, HSC-3 and OEC-M1 oral cancer cells (Figs. 1A and S1A, B). CoCl_2_ treatment is a well-established chemical hypoxia approach because CoCl_2_ alleviates HIF-1α degradation [39–41]. The results further showed that hypoxia activated Lon and the initiation of autophagy in a time- (Figs. 1B, C and S1C) and dose-dependent manner (Fig. S1D). We found that HIF-1α-Lon expression and the initiation proteins of autophagy, ULK1, ULK1-S555 phosphorylation, ATG13, and FIP200, were activated under hypoxia (Figs. 1B and S1C); the chronological activation of autophagy was showed by the HIF-1α-Lon-ULK1 axis (Fig. 1C). We also found that hypoxia treatment causes the phosphorylation of ULK1 downstream targets, ATG14 Serine 29 and Beclin1 Serine 15 (Fig. S1E, F), and the phosphorylation were reduced by the treatment of SBI-0206965, a potent ULK1 kinase inhibitor (Fig. S2A, B), which is supported by the recruitment of ATG14 and Beclin1 to the autophagosome initiation complex by ULK1 activation [42, 43].

Since mitophagy is induced by impaired mitochondria under stress and related to mitochondrial dynamics by fission or fusion [44, 45], we also found that the phosphorylation of Drp1 at serine 616 for mitochondrial fission was increased first and then decreased whereas fusion (p-DRP1 serine 637) was increased later under hypoxia (Fig. S2C). These supported the ULK1 activation for the selective mitophagy response under hypoxia stress. To confirm that hypoxia-induced ULK1 activation is involved in the selective autophagic response under hypoxic stress, the autophagic assay was performed using bafilomycinA1 (BafA1) treatment, a potent inhibitor of autophagosome and lysosome fusion (Fig. 1D–G). The results showed that LC3B-II levels were increased under hypoxia treatment and were dramatically increased in threefold upon BafA1 treatment (Fig. 1D, E). To quantify the mitophagy flux under hypoxia, we transiently transfected mito-QC (a tandem GFP-mCherry fusion reporter [46]) in cancer cells before hypoxia treatment. To validate that the mCherry-only puncta are mitolysosomes, a degraded mitochondrial component within lysosomes, we measured the co-localization signals of mCherry puncta and LAMP1, a lysosomal marker protein. The results showed that mCherry entry into lysosomes was inhibited by BafA1 treatment. We found that number of mitolysosomes (mCherry-only in lysosomes) was threefold increased under hypoxia treatment compared with the control, and more than 80% of mCherry puncta were inhibited in LAMP-positive lysosomes upon BafA1 treatment (Fig. 1F, G). These data concluded that hypoxia induces the activation of mitophagy and activates Lon upregulation and the ULK1 downstream autophagy signaling concurrently.

### Mitochondrial Lon is significant for ULK1-induced mitophagy under hypoxia

**Fig. 2:**
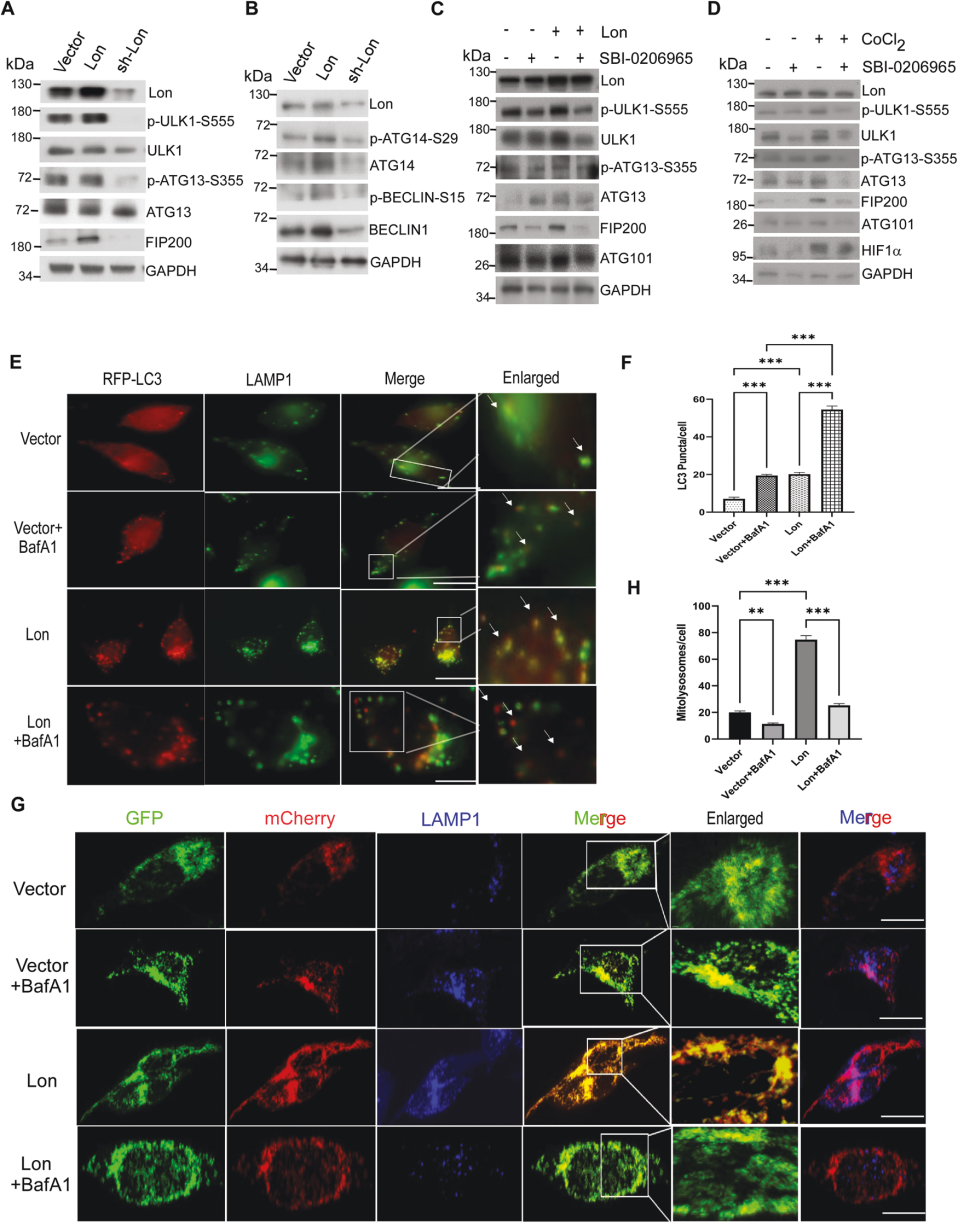
Mitochondrial Lon is required for ULK1-mediated mitophagy under hypoxia. **A**, **B** HCT-15 cells were transiently transfected with the plasmids encoding Lon or Lon-shRNA. Cell lysates were analyzed by immunoblotting using the indicated antibodies. GAPDH as the loading control. **C** HCT-15 cells transfected with the plasmids encoding Lon or empty were treated with SBI-0206965 (20 μM for 6 h) or not. Cell lysates were analyzed by immunoblotting using the indicated antibodies. GAPDH as the loading control. **D** HCT-15 cells were treated with CoCl_2_ (200 μM for 18 h) or not in the presence or absence of SBI-0206965 (20 μM for 6 h). Cell lysates were analyzed by immunoblotting using the indicated antibodies. GAPDH as the loading control. **E**, **F** Mitochondrial Lon induces lysosome-mediated autophagy. **E** Mitochondrial Lon-induced lysosome-mediated autophagy were verified by immunofluorescence. HCT-15 cells were transfected with the plasmids encoding Lon or empty and treated with BafilomycinA1 (100 nM for 6 h) or not. The cells were fixed and immunostained by RFP-LC3B (red) and anti-LAMP1 (green) antibodies. Scale bars = 50 μm. **F** Mitochondrial Lon-induced lysosome-mediated autophagy were quantified by LC3B puncta/cell detection according to the immunofluorescence data in E. LC3B puncta/cell was quantified by selecting more than 50 cells per condition. *n* = 3 biological replicates. The error bars shown in the panel represent the standard deviation from three independent experiments. ***p < 0.001. **G**, **H** Mitochondrial Lon induces lysosome-mediated mitophagy. **G** Mitochondrial Lon-induced lysosome-mediated mitophagy were verified by confocal immunofluorescence. HCT-15 cells transiently expressing mito-QC were transfected with the plasmids encoding Lon or empty and treated with BafilomycinA1 (100 nM for 6 h) or not. The cells were fixed and immunostained by GFP (mitochondria, green), mCherry (mitolysosome, red), and anti-LAMP1 (lysosome, blue) antibodies. Scale bars = 10 μm. **H** Mitochondrial Lon-induced lysosome-mediated mitophagy were quantified by mcherry signals according to the immunofluorescence data in F. *n* = 3 biological replicates. The error bars shown in the panel represent the standard deviation from three independent experiments. ***p < 0.001.

To explore the role of mitochondrial Lon in regulation of autophagy, we first examined the expression level of ULK1 and its autophagy complex proteins when cells were transfected with the plasmid pCDNA3-Lon or Lon-shRNA (Fig. 2). We found that the initiation proteins of autophagy were increased and ULK1 downstream target proteins were significantly activated upon Lon overexpression whereas the initiation proteins of autophagy were inhibited in the Lon-shRNA HCT-15 cells (Fig. 2A, B) and in OEC-M1 oral cancer cells (Fig. S3A). To confirm ULK1 is important for Lon-induced mitophagy, we treated the cells with the inhibitor SBI-0206965. We found that hypoxia- and Lon-induced autophagy signaling is inhibited by ULK1 inhibitor (Fig. 2C, D). Similar results were observed in Lon-induced activation of ULK1 targets (Fig. S3B). Since mitochondrial Lon has been proved to induce ROS production [31], we confirmed that hypoxia- and Lon-induced autophagy signaling was inhibited by NAC, a ROS scavenger (Fig. S3C, D). These data certainly suggested the crucial involvement of Lon in the regulation of ULK1 function in the early stage of autophagosome biogenesis.

To confirm that Lon-induced ULK1 activation is involved in autophagic flux, the autophagic assay was performed using bafilomycinA1 (BafA1) treatment, a potent inhibitor of autophagosome and lysosome fusion. The results showed that LC3B-II levels and RFP-LC3 puncta were increased under Lon overexpression and were dramatically increased upon BafA1 treatment (Figs. 2E, F and S3E). Similarly, mitophagic flux quantification was performed under condition of Lon overexpression using mito-QC reporter and LAMP1 co-localization. As expected, we observed that mCherry puncta in LAMP1 positive lysosomes were increased upon Lon upregulation and inhibited upon BafA1 treatment (Fig. 2G, H). Altogether, these findings indicate that Lon is involved in the early event of mitophagy signaling and may stabilize specific ULK1 kinase complex for the mitophagy turnover.

### Mitochondrial Lon chaperone activity contributes to the stability of ULK1 complex for the mitophagy activation

**Fig. 3:**
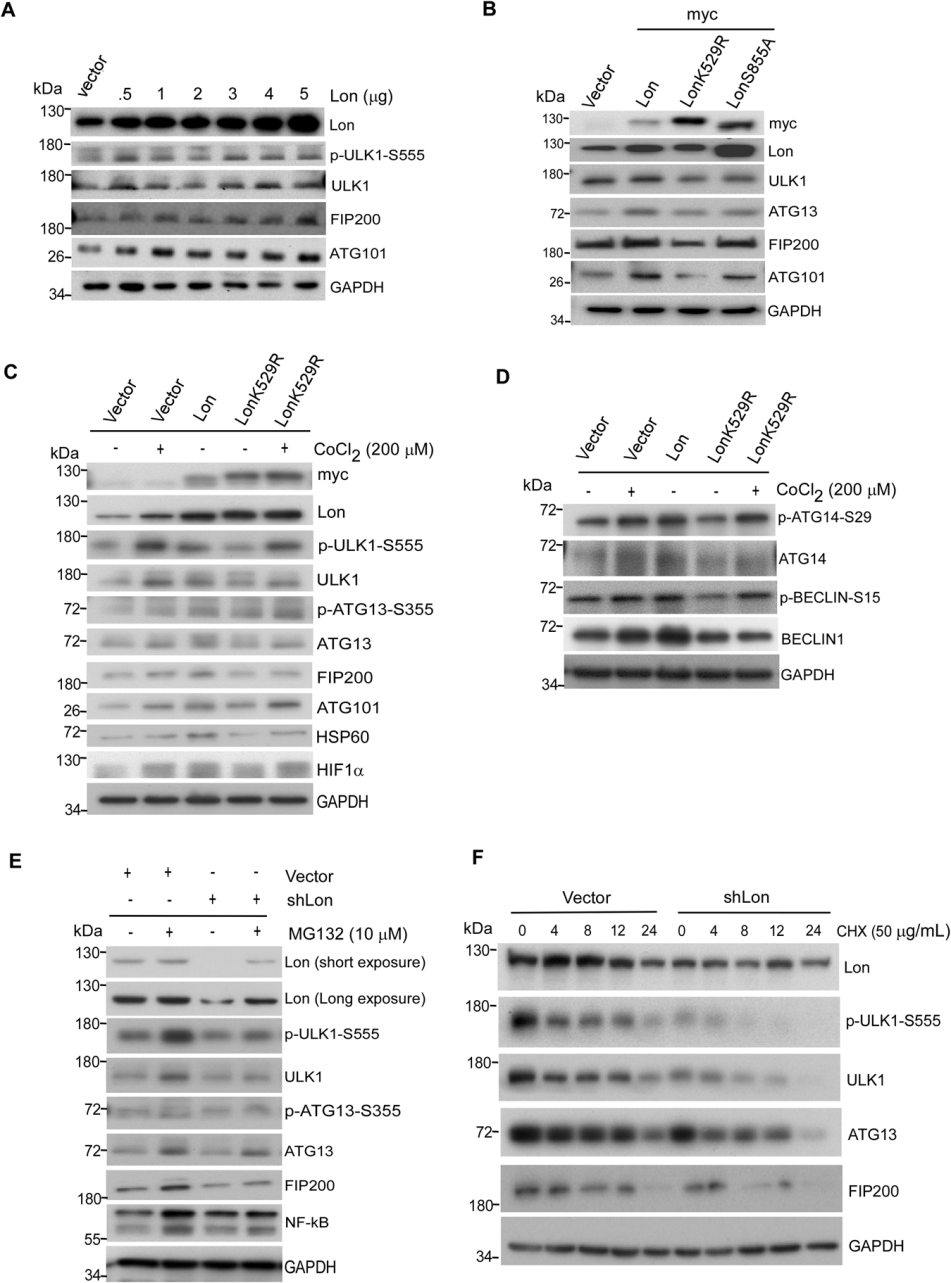
Mitochondrial Lon chaperone activity contributes to the stability of ULK1 complex for the mitophagy activation. **A** HCT-15 cells were transfected with the plasmids encoding pcDNA3-Lon in different concentrations (0.5–5 μg). Cell lysates were analyzed by immunoblotting using the indicated antibodies. GAPDH as the loading control. **B** HCT-15 cells were transfected with the plasmids encoding myc-Lon, myc-LonK529R (ATPase mutant), or myc-LonS855A (protease mutant). Cell lysates were analyzed by immunoblotting using the indicated antibodies. GAPDH as the loading control. **C**, **D** HCT-15 cells were transfected with the plasmids encoding empty, myc-Lon, or myc-LonK529R in the presence or absence of CoCl_2_ treatment (200 μM for 18 h). Cell lysates were analyzed by immunoblotting using the indicated antibodies. GAPDH as the loading control. **E** HCT-15 cells transfected with the plasmids encoding Lon-shRNA or empty were treated with or without MG132 (10 μM for 6 h). Cell lysates were analyzed by immunoblotting using the indicated antibodies. GAPDH as the loading control. **F** HCT-15 cells transfected with the plasmids encoding Lon-shRNA or empty vector were treated with or without Cycloheximide (50 µg/mL) for the indicated time course. Cell lysates were analyzed by immunoblotting using the indicated antibodies. GAPDH as the loading control.

To confirm mitochondrial Lon influences the stability of ULK1 complex and its kinase activity for the mitophagy activation, we overexpressed Lon in cells with pCDNA3-Lon plasmid. Mitochondrial Lon protein level was increased with simultaneous increase in ULK1/FIP200/ATG101 complex (Fig. 3A). This depicts that Lon chaperone function may influence the ULK1 stability and its kinase-dependent function of mitophagy. To establish the role of Lon chaperone activity in the ULK1-dependent mitophagy, we overexpressed myc-Lon-WT, myc-Lon-K529R, or myc-Lon-S855A in cells where empty vector used as a control. The K529R mutant removed the conserved lysine residue in the Walker A motif of the ATPase domain [47] whereas the S855A mutant removed the catalytic serine in the protease domain [21]. The results indicated that the protein level of ULK1 and its complex were significantly increased upon WT Lon expression but decreased upon the LonK529R mutant overexpression. The protease mutant LonS855A has shown no significant changes compared with the WT overexpression (Fig. 3B). To confirm the role of Lon chaperone activity in the hypoxia-induced mitophagy, we performed the rescue experiment using the LonK529R mutant and CoCl_2_ treatment. Consistently, hypoxia treatment and Lon overexpression induced an increase in ULK1/FIP200/ATG101 complex and the activation of autophagy (Fig. 3C). We found that the LonK529R mutant suppressed the phosphorylation of ULK1 and the stability of ULK1 complex. However, hypoxia treatment restored the protein level and activation of ULK1 complex in LonK529R-overexpressed cells (Fig. 3C). Hsp60, a mitochondrial chaperone, was used as a positive control that is a member of Lon-mtHsp60-mtHSP70 complex [23, 24]. Similar results were observed in the activation of ULK1 downstream molecules for phagophore initiation, ATG14 and Beclin1 (Fig. 3D). In addition, we confirmed that the activation of ULK1 complex could be rescued upon overexpression of either Flag-ULK1 (Fig. S4A) or myc-Lon (Fig. S4B) expression upon the LonK529R mutant overexpression. Since the p32/C1QBP stabilizes the ULK1 against proteasome-mediated degradation [48], we tried to examine whether chaperone Lon protects the ULK1 complex from proteasome degradation. Thus, we used MG132, a well-known proteasome inhibitor, and cycloheximide (CHX), a protein synthesis inhibitor, to treat the Lon-shRNA expressing cells. The results showed that Lon-shRNA treatment resulted in depletion of the ULK1 complex; however, MG132 treatment rescued the protein level of the ULK1 complex in the Lon-shRNA expressing cells (Fig. 3E). To exclude the effect of MG132 on Lon protein level, we treated CHX in the Lon-shRNA expressing cells. The CHX experiment showed that Lon-shRNA further reduced the protein level of the ULK1 complex under the CHX treatment (Fig. 3F) and Lon overexpression rescued the protein level of the ULK1 complex under the same condition (Fig. S4C). These experiments exclude the effect of the treatment on Lon protein level and confirm the interaction between Lon and the ULK1 complex, which conclude that Lon maintains the stability of ULK1 complex. Collectively, these data ensure the specificity and involvement of Lon chaperone activity in stabilizing the ULK1 complex for the mitophagy activation.

### Lon and FUNDC1-ULK1 complex accumulate at ER-mitochondria tethering sites upon hypoxia

**Fig. 4:**
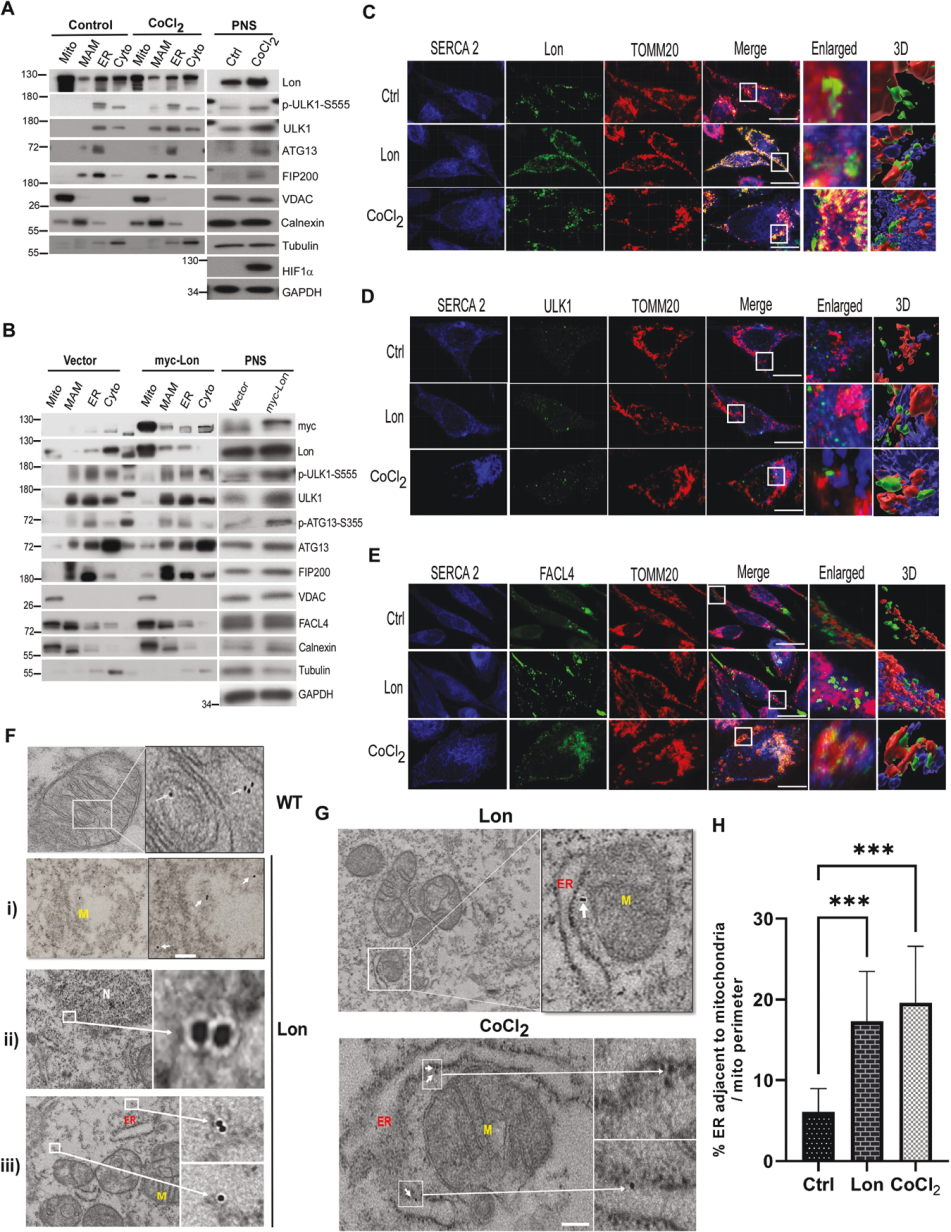
Lon and ULK1 complex accumulates at ER-mitochondria tethering sites in response to hypoxia. **A**, **B** Lon and ULK1 complex accumulates at ER-mitochondria contact (EMC) sites under hypoxia. HCT-15 cells treated with or without CoCl_2_ (200 μM for 18 h) (**A**) or transfected with the plasmids encoding myc-Lon (**B**) were used to perform subcellular fractionation experiment. Cell lysates were analyzed by immunoblotting using the indicated antibodies. GAPDH as the loading control. Mito mitochondria, MAM mitochondria associated membranes, ER endoplasmic reticulum, cyto cytosol, PNS post nuclear supernatant. **C**–**E** Accumulation of Lon and ULK1 at the EMC/MAM was verified by confocal immunofluorescence. HCT-15 cells were transfected with the plasmids encoding Lon or treated with CoCl_2_ (200 μM for 18 h) or not. The cells were fixed and immunostained by anti-Lon (green) (**C**), anti-ULK1 (green) (**D**), anti-FACL4 (ER and MAM, green) (**E**), anti-SERCA-2 (ER, blue), and anti-TOMM20 (mitochondria, red) antibodies. Scale bars = 10 μm. **F**–**H** Localizaton of Lon at the EMC/MAM was verified by transmission electron microscopy (TEM). HCT-15 cells were treated with CoCl_2_ (200 μM for 18 h). The cells were fixed and and immunostained by anti-Lon and immunogold labeling antibodies. The immunogold electron micrographs showed Lon in (i) damaged mitochondria (M), (ii) ER around Nucleus (N), (iii) Cytosol (**F**) and the ER-mitochondria tethering sites (**G**). ER: endoplasmic reticulum. Scale bars: 100 nm. Quantitation of the percentage of ER adjacent to mitochondria in both CoCl_2_ and Lon expressed HCT-15 cells and compared with control (**H**). The percentage was normalized by total ER and mitochondrial perimeter (*n* = 36 field for each condition). *n* = 3 biological replicates. The error bars shown in the panel represent the standard deviation from three independent experiments. ***p < 0.001.

The ULK1 complex has been reported the function of omegasome formation during initiation of autophagy [49] and further translocation of ULK1 complexes that binds FUNDC1 in the EMC region [50–52]. The EMC plays a significant role in the autophagosome biogenesis during autophagy/mitophagy. In addition, mitochondrial Lon was identified as a novel EMC protein despite the detail function still remains unknown [33]. We thus speculated that, under hypoxia, the EMC is the important region for interaction between Lon and FUNDC1-ULK1 complex to initiate the mitophagy. Firstly, to confirm the accumulation of Lon and ULK1 complex in the EMC under hypoxia, we analyzed the subcellular distribution of Lon and ULK1 complex in both normal and hypoxia treated cells by Percoll density-gradient centrifugation. Different fractions were identified using the following organelle markers: VDAC1 (mitochondria and EMC/MAM), calnexin, FACL4 (ER and EMC/MAM), and Tubulin (Cytosol) [53, 54]. We found that both ULK1 and Lon accumulates at the EMC sites in response to hypoxia, although some amount of Lon can be also detected in the EMC under normoxia condition (Fig. 4A). This suggests the possibility that Lon and ULK1 complex association is established strictly in the EMC under hypoxia. To further prove the translocation of Lon to the EMC under hypoxia, we repeated the experiment of subcellular fractionation using the cells overexpressing myc-Lon and the empty vector as a control. Similarly, the accumulation of Lon was significantly increased in the EMC fraction upon Lon overexpression only, and the accumulation of ULK1 complex only in the EMC fraction was substantially increased upon Lon overexpression (Fig. 4B). To validate the interaction between Lon and ULK1 in the EMC under hypoxia, we performed the co-localization experiment by immunofluorescence microscopy imaging to show the association of Lon with ULK1, TOMM20 (mitochondria), SERCA-2 (an ER transporter), and FACL4 (ER and EMC). The results confirmed that mitochondrial Lon can co-localize with outer membrane protein TOMM20 (Fig. 4C). We then found that mitochondrial Lon is also able to co-localize with SERCA-2 under hypoxia (Fig. 4C), suggesting that the accumulation of Lon exits in the EMC site under hypoxia. We next validated the interaction between TOMM20/Lon and ULK1 in the ER (Fig. 4D) and the interaction between SERCA-2 and FACL4 (Fig. 4E) under hypoxia. This observation was confirmed by the interaction between TOMM20/Lon and FACL4 under hypoxia (Fig. 4E). These findings reveal the association of Lon and ULK1 at the EMC sites in response to hypoxia. To confirm this, we used the gold-labeled immunostaining TEM to detect Lon localization under Lon overexpression and hypoxia. We observed that gold-labeled Lon was detected in the mitochondria (WT and Lon, i, Fig. 4F) and ER (ii, Fig. 4F) and cytosol (iii, Fig. 4F) upon hypoxia. Consistently, the Lon was detected at the ER-mitochondria contact sites (Fig. 4G), and the EMC sites were increased (Fig. 4H) under both Lon overexpression and hypoxia treatment. Altogether, our results supported the idea that Lon accumulation in the EMC under hypoxia is important for the interaction and recruitment of the FUNDC1-ULK1 complex for the mitophagy initiation.

### Mitochondrial Lon interacts with and stabilizes FUNDC1-ULK1 complex under hypoxia

**Fig. 5:**
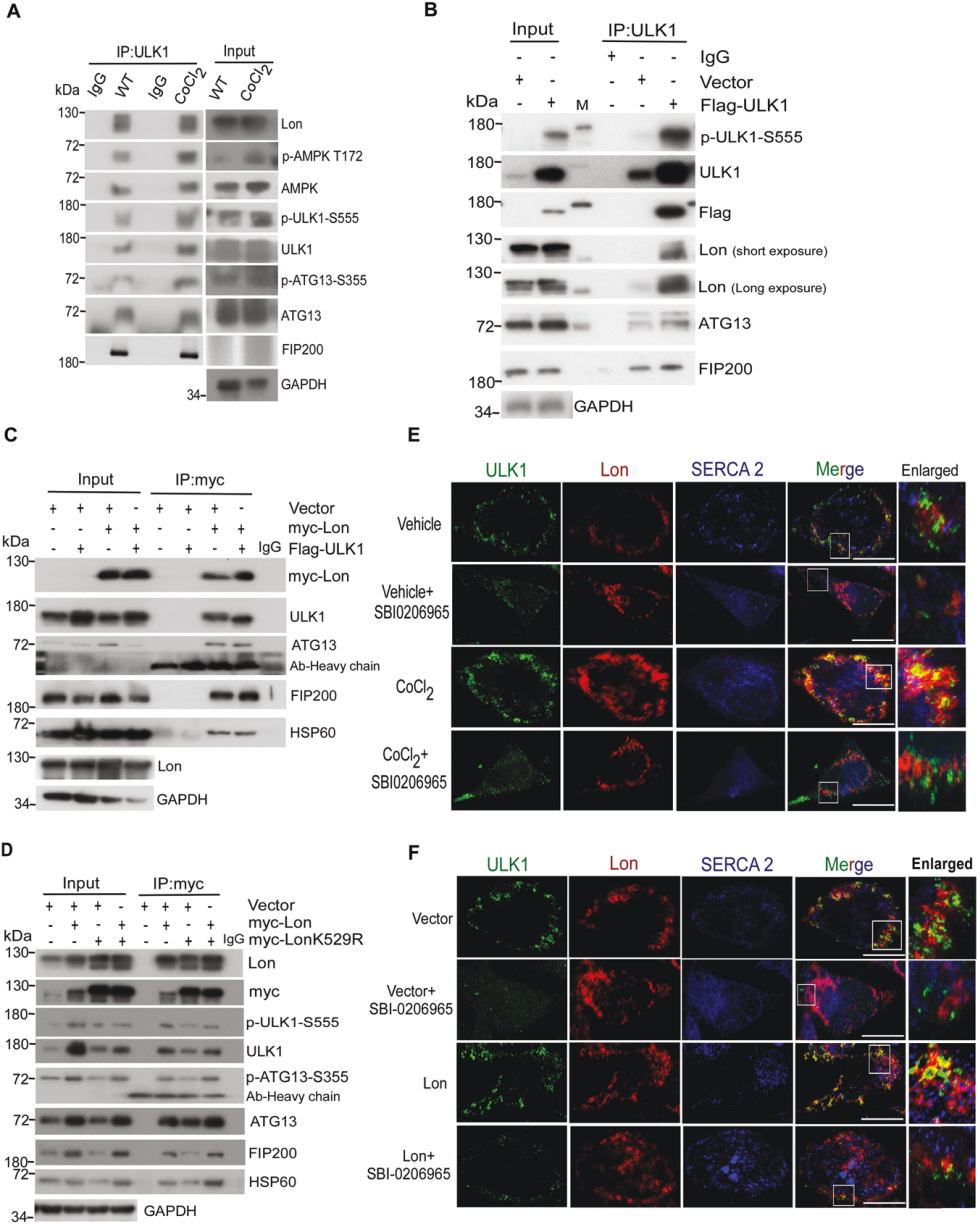
Mitochondrial Lon interacts with and stabilizes ULK1 complex under hypoxia. **A**–**D** Mitochondrial Lon interacts with ULK1 shown by co-immunoprecipitation. **A** HCT-15 cells were treated with CoCl_2_ followed by co-immunoprecipitation with anti-ULK1. Whole cell lysates from HCT-15 cells treated with CoCl_2_ (200 μM for 18 h) were immunoprecipitated with anti-ULK1 antibodies. The immunoprecipitation complex was analyzed by Western blotting using the indicated antibodies. IP, immunoprecipitation. **B** Whole cell lysates from HCT-15 cells transfected with the plasmids encoding Flag-ULK1 or vector were immunoprecipitated with anti-ULK1 antibodies. The immunoprecipitation complex was analyzed by Western blotting using the indicated antibodies. **C** Whole cell lysates from HCT-15 cells transfected with the plasmids encoding myc-Lon and Flag-ULK1 were immunoprecipitated with anti-myc antibodies. The immunoprecipitation complex was analyzed by Western blotting using the indicated antibodies. **D** Whole cell lysates from HCT-15 cells transfected with the plasmids encoding myc-Lon or myc-LonK529R were immunoprecipitated with anti-myc antibodies. The immunoprecipitation complex was analyzed by Western blotting using the indicated antibodies. **E**, **F** Mitochondrial Lon interacts with ULK1 shown by immunofluorescence. **E** The interaction of Lon with ULK1 was enhanced by ULK1 activity under hypoxia. HCT-15 cells treated with or without CoCl_2_ (200 μM for 18 h) in the presence or absence of SBI-0206965 (20 μM for 6 h) were immunostained by anti-ULK1 (green) and anti-Lon (red) following image capturing by immunofluorescence microscopy. DAPI was used for nuclear staining. Scale bars, 50 μm (*n* = >50 cells/condition and 3 biological replicates). **F** The interaction of Lon with ULK1 was enhanced by ULK1 activity. HCT-15 cells transfected with the plasmids encoding Lon or empty in the presence or absence of SBI-0206965 (20 μM for 6 h) were immunostained by anti-ULK1 (green) and anti-Lon (red) following image capturing by immunofluorescence microscopy. DAPI was used for nuclear staining. Scale bars, 50 μm (*n* = >50 cells/condition and 3 biological replicates).

The chaperone function of Lon may be associated with the binding to ULK1 and its interacting proteins. To prove this, we performed co-immunoprecipitation (co-IP) experiments with either Lon or ULK1 under hypoxia treatment. The results revealed that endogenous Lon associates with endogenous ULK1 and its complex, ATG13 and FIP200 (Figs. 5A and S5A). Moreover, we found that endogenous Lon was immunoprecipitated by ULK1 antibody upon transfection of Flag-ULK1, and ATG13 and FIP200 were considered as the positive control (Fig. 5B). Consistently, the ULK1 complex was associated with myc-Lon using anti-myc antibody upon co-transfection of Flag-ULK1 and myc-Lon (Fig. 5C), and myc-Lon was immunoprecipitated by using anti-Flag antibody (Fig. S5B). Next, we examined whether the chaperone activity of Lon is critical for the association between Lon and the ULK1 complex. Hence, we individually transfected myc-Lon or myc-LonK529R into cells and the ULK1 complex was further immunoprecipitated with anti-myc antibody. We found that the endogenous ULK1 complex proteins were immunoprecipitated by myc-Lon, whereas the interaction between Lon and ULK1 complex was significantly abolished using transfection of myc-LonK529R (Fig. 5D). These data clearly show that ATPase activity of mitochondrial Lon is required for the association with the ULK1 complex. Consistently, the immunofluorescence results showed that colocalization between Lon and ULK1 was enhanced in both hypoxia treatment (Fig. 5E) and Lon overexpression (Fig. 5F) but the localization was strictly inhibited upon treatment with SBI-0206965, the inhibitor of ULK1 kinase (Fig. 5E, F). These data indicate that ATPase activity of mitochondrial chaperone Lon is required for the association with the ULK1 complex, and the ULK1 kinase activity further contributes the stability of the association.

**Fig. 6:**
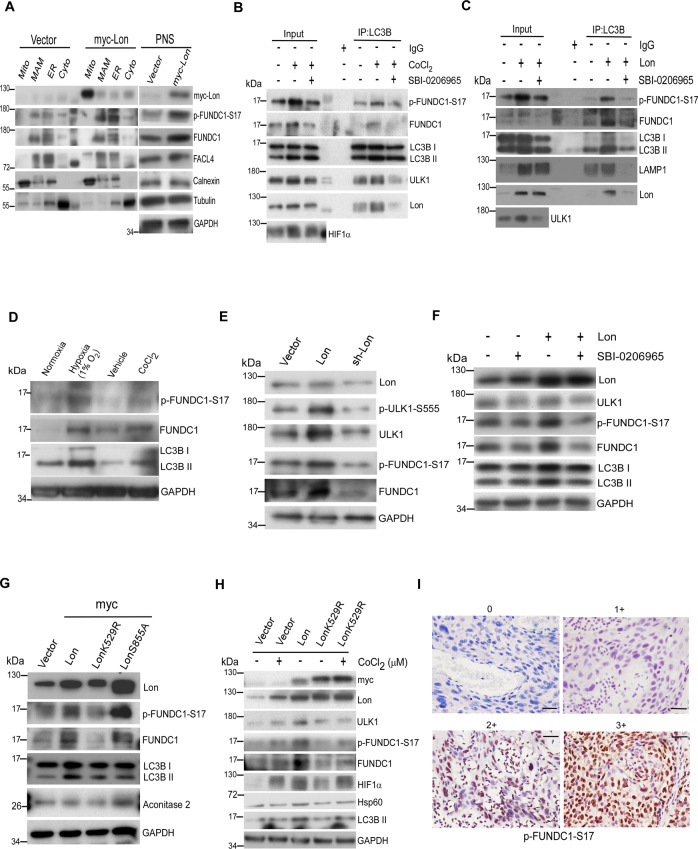
FUNDC1-Ser17 phosphorylation by ULK1 kinase at the EMC/MAM is important for the Lon-induced mitophagy. **A** HCT-15 cells transfected with the plasmids encoding myc-Lon were used to perform subcellular fractionation experiment. Cell lysates were analyzed by immunoblotting using the indicated antibodies. GAPDH as the loading control. Mito mitochondria, MAM mitochondria associated membranes, ER endoplasmic reticulum, cyto cytosol, PNS post nuclear supernatant. **B** HCT-15 cells were treated with CoCl_2_ (200 μM for 18 h) or not in the presence or absence of SBI-0206965 (20 μM for 6 h). Whole cell lysates from the treated HCT-15 cells were immunoprecipitated with anti-LC3B antibodies. The immunoprecipitation complex was analyzed by Western blotting using the indicated antibodies. **C** HCT-15 cells transfected with the plasmids encoding Lon or empty vector were treated with SBI-0206965 (20 μM for 6 h) or not. Whole cell lysates from the treated HCT-15 cells were immunoprecipitated with anti-LC3B antibodies. The immunoprecipitation complex was analyzed by Western blotting using the indicated antibodies. **D** HCT-15 cells were treated with or without CoCl_2_ (200 μM for 18 h) or hypoxia exposure (1% O_2_). Cell lysates were analyzed by immunoblotting using the indicated antibodies. GAPDH as the loading control. **E** HCT-15 cells were transfected with the plasmids encoding Lon or Lon-shRNA. Cell lysates were analyzed by immunoblotting using the indicated antibodies. GAPDH as the loading control. **F** HCT-15 cells transfected with the plasmids encoding Lon or empty were treated with SBI-0206965 (20 μM for 6 h) or not. Cell lysates were analyzed by immunoblotting using the indicated antibodies. GAPDH as the loading control. **G** HCT-15 cells were transfected with the plasmids encoding myc-Lon, myc-LonK529R (ATPase mutant), or myc-LonS855A (protease mutant). Cell lysates were analyzed by immunoblotting using the indicated antibodies. GAPDH as the loading control. **H** HCT-15 cells were transfected with the plasmids encoding empty, myc-Lon, or myc-LonK529R in the presence or absence of CoCl_2_ treatment (200 μM for 18 h). Cell lysates were analyzed by immunoblotting using the indicated antibodies. GAPDH as the loading control. **I** Immunohistochemical analysis of p-FUNDC1-S17 expression in OSCC patients. Representative immunohistochemical staining of p-FUNDC1-S17 was performed using paraffin-embedded sections of OSCC. The representative intensity of immunostaining was classified as four levels: negative staining intensity (0) and positive staining intensity, including weak (1+), median (2+), and strong (3+) staining. Microscopic magnification, ×400. Scale bar, 50 μm.

We further examined the presence of FUNDC1 and p-FUNDC1-S17 in mitochondria and the EMC by analyzing the subcellular fractions in cells. The results indicated that both FUNDC1 and p-FUNDC1-S17 were accumulated in the EMC fraction under Lon overexpression than the vector control (Fig. 6A). To further examine the function of Lon-ULK1-FUNDC1 complex in mitophagy under hypoxia, we detected the interaction between FUNDC1-S17 and LC3B by the immunoprecipitation experiment using anti-LC3B antibody. The results confirmed that Lon shows a strong interaction with ULK1-FUNDC1-S17-LC3B complex upon hypoxia treatment. The interaction between FUNDC1-S17 and LC3B was significantly abolished upon the treatment of ULK1 inhibitor SBI-0206965 under the same condition (Fig. 6B). Consistently, the interaction between FUNDC1-S17 and LC3B was significantly abolished upon SBI-0206965 treatment under Lon overexpression (Fig. 6C). FUNDC1 and LAMP1 were used as positive controls. Collectively, these data indicate that Lon interaction is involved in the FUNDC1-ULK1-dependent mitophagy at the EMC upon hypoxic condition.

### FUNDC1-S17 phosphorylation by ULK1 kinase is important for the Lon-induced mitophagy

FUNDC1 (FUN14 domain-containing protein-1), an outer mitochondrial membrane protein, is a parkin-independent hypoxia-specific mitophagy receptor that binds to LC3 [16, 55, 56]. ULK1 kinase phosphorylates FUNDC1 at Serine 17 and promotes the interaction between FUNDC1 and LC3 in response to hypoxia [57]. We next investigated the role of mitochondrial Lon in the mitophagy induction through enhancing the FUNDC1 phosphorylation by ULK1 under hypoxia. The results found that p-FUNDC1-S17 was increased along with ULK1 activity under hypoxia (Figs. 6D and S3F). We further evaluated the role of Lon in the regulation of p-FUNDC1-S17 phosphorylation. We found that Lon upregulation induces extensive increase in protein and phosphorylation level of FUNDC1-S17 whereas the effect has been diminished upon knockdown of Lon (Fig. 6E), suggesting the significance of Lon in regulating the mitophagy through ULK1. We confirmed that the mitophagy induced by hypoxia and Lon was inhibited the treatment of SBI-0206965, an ULK1 kinase inhibitor (Figs. 6F and S3G). We then examined the role of Lon chaperone activity in the p-FUNDC1-S17-dependent mitophagy. Consistently, Lon increased the total FUNDC1 and the phosphorylation status of FUNDC1-S17 and its downstream of the LC3B activation, but not in the ATPase mutant LonK529R (Fig. 6G). The protease mutant of Lon (Lon-S855A) still showed a significant increase in both FUNDC1/FUNDC1-Ser17 phosphorylation and LC3B. Furthermore, the inhibition of FUNDC1-S17 phosphorylation and LC3B II level by the LonK529R mutant could be rescued by hypoxia treatment (Fig. 6H), suggesting that the chaperone property of Lon is involved in the FUNDC1-dependent mitophagy upon hypoxic condition. These data suggest that ATPase activity of Lon is important for the binding to FUNDC1 and the ULK1 stability, and kinase activity of ULK1 in mitophagy under hypoxia. Collectively, these data indicate that the chaperone activity of Lon is involved in the FUNDC1-ULK1-dependent mitophagy at the EMC upon hypoxic condition.

To associate the clinical significance of Lon-induced mitophagy in cancer progression, we examined whether the FUNDC1-S17 phosphorylation regulated by Lon is clinically relevant in cancer, for example oral cancer. The expression pattern of Lon and p-FUNDC1-S17 in 92 samples of tumor tissues from OSCC patients was determined by Immunohistochemistry (IHC) analysis. The clinicopathological characteristics of the patients in this study are as described in our previous results [23]. Representative samples defined negative, weak, median, and strong staining of Snail are shown (IHC level 0 to 3+, Fig. 6I). The association between Lon and p-FUNDC1-S17 expression in OSCC tissues was tested in the contingency table using Fisher’s exact test. The result showed that p-FUNDC1-S17 expression showed a significant correlation with Lon expression (*P* = 0.01063, Table 1). Consistently, the correlation between Lon and p-FUNDC1-S17 expression is statistically significant by Spearman’s rank test (*P* = 0.00239, Table 1). Taken together, these data indicate a direct correlation between the axis of Lon-ULK1 and mitophagy on cell survival and cancer progression under hypoxia condition in the tumor microenvironment.

**Table 1:** The contingency table shows a positive association between Lon and p-FUNDC1-S17, based on 92 OSCC patients with Lon/p-FUNDC1-S17 protein staining.

		Lon			Fisher, *P*	Spearman’s
		None/weak	Median	Strong		Rank correlation (ρ, *P*)
p-FUNDC1 (S17)	None/Weak	29	18	22	0.01063	0.313, 0.00239
	Median	3	6	8	–	–
	Strong	0	0	6	–	–

### Mitochondrial Lon in the EMC depends on the interaction with NCLX to increase the cytosolic calcium levels and activate FUNDC1-ULK1 mitophagy under hypoxia

**Fig. 7:**
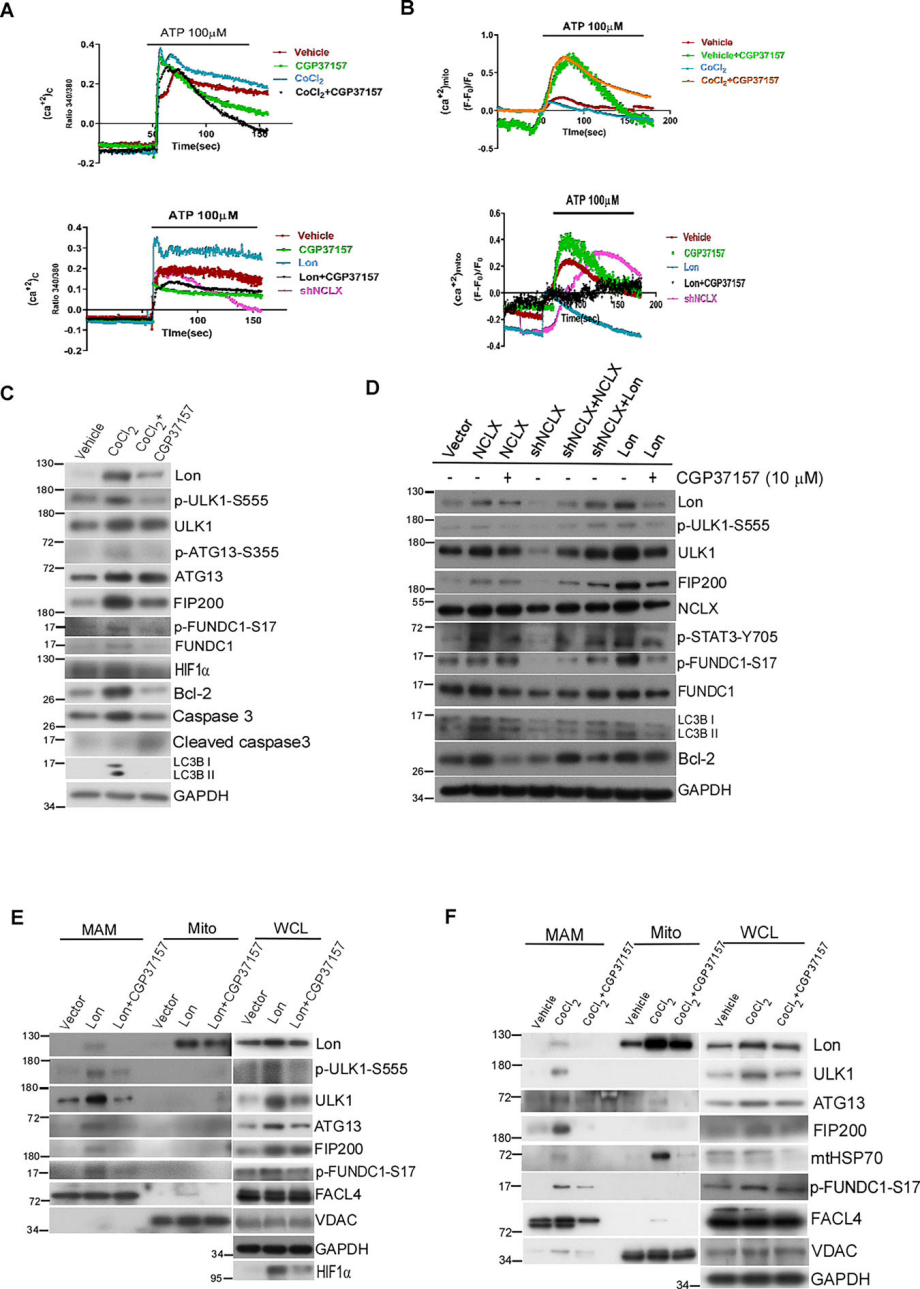
Lon binds with mitochondrial Na^+^/Ca^2+^ exchanger to promote FUNDC1-ULK-mediated mitophagy in the EMC/MAM site. **A**, **B** Ca^2+^_mito_ (mt-lar-GECO) and Ca^2+^_cyto_ (Fura-2 AM) were measured by live-cell microscopy in OEC-M1 treated with CoCl_2_ (300 µM-18h) or transfected with Lon in presence or absence of CGP37157 (10 µM-4h). ATP (100 µM) was used as an agonist and further measured and analyzed (*n* = 3). **C** OEC-M1 cells were treated with CoCl_2_ (300 µM-18 h) in the presence or absence of CGP37157 (10 µM-4 h). Cell lysates were analyzed by immunoblotting using the indicated antibodies. GAPDH as the loading control. **D** OEC-M1 cells were transfected with Vector, Lon, NCLX (48 h) in the presence or absence of CGP37157 (10 µM-4h) and shNCLX transfected OEC-M1 cells were co-transfected with NCLX and Lon plasmids and incubated for 48 h. Cell lysates were analyzed by immunoblotting using the indicated antibodies. GAPDH as the loading control. **E**, **F** Lon and ULK1 complex accumulation at EMC/MAM sites were abolished upon NCLX inhibition under hypoxia. OEC-M1 cells transfected with the plasmids encoding myc-Lon (**E**) or treated with CoCl_2_ (200 μM for 18 h) (**F**) in presence or absence of CGP37157 (10 µM-8h) were used to perform subcellular fractionation experiments. Cell lysates were analyzed by immunoblotting using the indicated antibodies. GAPDH as the loading control. Mito mitochondria, EMC ER-mitochondria contact sites, MAM mitochondria associated membranes, WCL whole cell lysate.

**Fig. 8:**
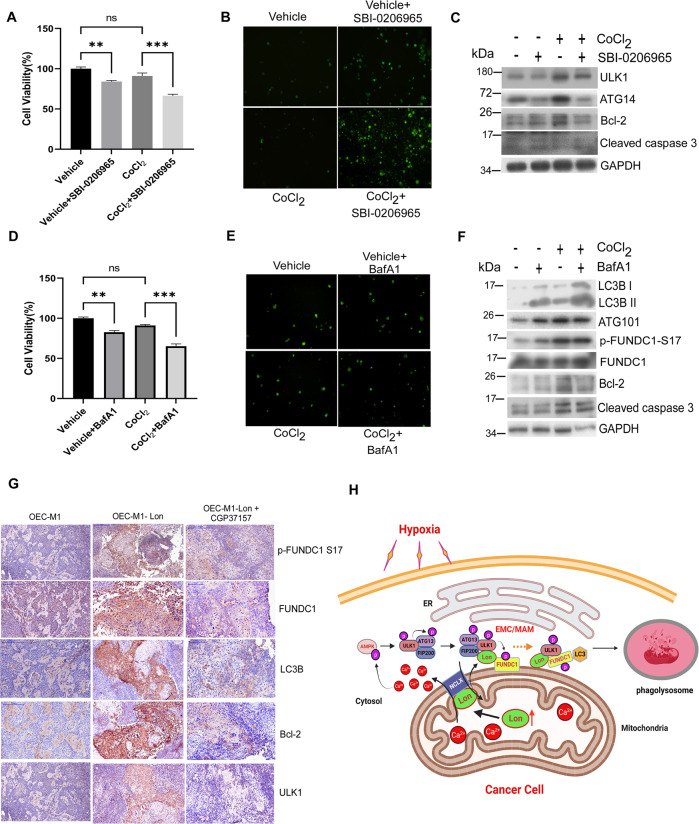
Lon-ROS-ULK1-FUNDC1 axis induced mitophagy benefits cell survival and tumorigenesis in vitro and in vivo. **A–C** HCT-15 cells were treated with CoCl_2_ (200 μM for 18 h) or not in the presence or absence of SBI-0206965 (20 μM for 24 h). The MTS assay for cell viability (**A**), fluorescence-based cleaved Caspase 3 apoptosis assay (**B**), and western blotting analysis (**C**) were performed. Immunoblots were obtained using the indicated antibodies. Scale bar, 100 μm (*n* = >50 cells/condition and 3 biological replicates). **D–F** HCT-15 cells were treated with CoCl_2_ (200 μM for 18 h) or not in the presence or absence of Bafilomycin A1 (100 nM for 24 h). The MTS assay for cell viability (**D**), fluorescence-based cleaved Caspase 3 apoptosis assay (**E**), and Western blotting analysis (**F**) were performed. Immunoblots were obtained using the indicated antibodies. Scale bar, 100 μm (*n* = >50 cells/condition and 3 biological replicates). **G** Immunohistochemical analysis of ULK1, p-FUNDC1 S17, FUNDC1, LC3B, and Bcl-2 expression in Lon expressed OEC-M1 tumor generated in BALB/C Nu mice treated with or without CGP37157. Representative immunohistochemical staining of respective targets was performed using paraffin-embedded sections of tumors collected. Microscopic magnification, ×400. Scale bar, 50 μm. **H** Scheme of the interaction between mitochondrial Lon and ULK1 complex at the EMC/MAM promotes mitophagy under hypoxia by stabilizing FUNDC1-ULK1 complex that depends on mitochondrial Na^+^/Ca^2+^ exchanger (NCLX). Upon hypoxia, Lon promotes FUNDC1-ULK1-mediated mitophagy at the EMC/MAM site, which is dependent on the binding with mitochondrial Na^+^/Ca^2+^ exchanger (NCLX). This interaction stabilized the FUNDC1-ULK1 at the EMC and initiated the mitophagy through the regulation of Ca^2+^ levels between mitochondria and cytosol. Lon-ULK1 phosphorylates FUNDC1 at S17, and Lon-ULK1-FUNDC1 axis promotes mitophagosome-lysosome fusion. EMC ER-mitochondria contact sites, MAM mitochondria associated membranes. The scheme of this study was created with BioRender.com.

We then tried to find out the mechanism of Lon translocation to EMC to interact the FUNDC1-ULK1 complex for the mitophagy initiation under hypoxia. The cytosolic Ca^2+^ was reported to activate the autophagy signaling, and the low energy activation of AMPK/ULK1-mediated autophagy was repressed after mitochondrial calcium accumulation through MCU overexpression [58]. In addition, we recently proved that mitochondrial Lon was associated with NCLX to activate cytosolic calcium-dependent PYK2-SRC-STAT3 signaling contributing to the cisplatin resistance [32]. We speculated that Lon translocation to interact the FUNDC1-ULK1 complex is through the interaction of Lon with NCLX for accumulating in the MAM and inducing the cytosolic calcium release. To prove this, we first monitored the cytosol and mitochondrial calcium levels under CoCl_2_ treatment and Lon overexpression using Fura-2 AM dye and mt-lar-GECO, respectively. The cytosolic calcium levels were significantly increased and mitochondrial calcium levels were decreased upon CoCl_2_ treatment and Lon overexpression (Fig. 7A, B). Indeed, the treatment of NCLX inhibitor, CGP37157, or shNCLX largely reversed the change in mitochondrial and cytosolic calcium levels induced by CoCl_2_ treatment and Lon overexpression (Fig. 7A, B). To confirm the regulation of autophagy by mitochondrial calcium, both CoCl_2_ treatment and Lon overexpression in OEC-M1 cells consistently activated the Lon-ULK1-FUNDC1-mediated mitophagy pathway, whereas NCLX inhibition significantly impaired the mitophagy activation (Fig. 7C, D). Lon and NCLX significantly increased the Lon-ULK1-FUNDC1 signaling whereas CGP37157 treatment and shNCLX abolished the Lon-ULK1-FUNDC1 activation (Fig. 7D). We then examined whether NCLX activity is important for the Lon accumulation in EMC to regulate ULK1-FUNDC1-mediated mitophagy. In consistent with Fig. 4A, B, MAM fraction was enriched with Lon and ULK1 complex in OEC-M1 cells including p-FUNDC1-S17 under both CoCl_2_ treatment and Lon overexpression; however, CGP37157 treatment strictly inhibited the Lon and ULK1 complex accumulation in theEMC which signifies the calcium role in regulating the mitophagy (Fig. 7E, F). These data were further validated through immunohistochemical staining of ULK1, p-FUNDC1 S17, FUNDC1, LC3B, and Bcl-2 in Lon-overexpressed OEC-M1 tumors from mice in the presence or absence of CGP37157 (Fig. 8G). Collectively, these data show the significance of NCLX activity in regulating the Lon-ULK1-FUNDC1-mediated mitophagy in the EMC.

### Lon-ROS-ULK1-FUNDC1 axis induced mitophagy benefits cell survival under hypoxia

Mitophagy as a regulator of mitochondria quality control is involved in regulation of mitochondria-mediated cell death. To address whether mitophagy induction by hypoxia/Lon augments cell survival in cancer cells, we determined the cell viability of cancer cells treated with the ULK1 inhibitor SBI-0206965 or lysosomal H + ATPase inhibitor bafilomycin A1 (BafA1). We first found that hypoxia treatment decreases the cell viability in a minimal range about below 10% compared with the control without treatment (Fig. 8A), which was in consistent with previous report [59]. However, inhibition of ULK1 by SBI-0206965 treatment made cells more susceptible to hypoxia treatment and exacerbated a decline in cell viability (Fig. 8A). The mechanism of lower cell viability upon ULK1 kinase inhibition was mediated by caspase 3-dependent apoptosis shown by fluorescence-based cleaved caspase 3 assay and immunoblotting (Fig. 8B, C), which were consistent with previous reports [60, 61]. Similarly, we confirmed the finding that the mechanism of low cell viability upon ULK1 kinase inhibition was mediated by caspase 3-dependent apoptosis under Lon overexpression. We found that Lon overexpression promotes cell viability by decreasing the cleaved caspase-3 and increasing the Bcl-2 expression (Fig. S6A–C), which is consistent with our previous reports [23, 24, 29]. This inhibition of apoptosis by Lon overexpression was reversed by SBI-0206965 treatment, an ULK1 inhibitor (Fig. S6A–C). To understand the mitophagy is critical for cell survival, we tested the cell susceptibility upon BafA1 treatment under hypoxia treatment. The results showed that BafA1 treatment caused cells to be more susceptible to hypoxia treatment and exacerbated a decline in cell viability (Fig. 8D). In contrast, however, we found that although the cell susceptibility was increased after BafA1 treatment, the mechanism of lower cell viability was not mediated by caspase 3-dependent apoptosis (Fig. 8E, F). The similar results showed that Lon-induced cell survival through the FUNDC1-S17 phosphorylation and mitophagy and this increase in survival by Lon overexpression was reversed by BafA1 treatment (Figs. S3E and S6D, E); however, the mechanism by BafA1 was not mediated by caspase 3-dependent apoptosis (Fig. S6E). These data suggested that cell survival through mitophagy maybe involved in different types of cell death including caspase-dependent and -independent mechanisms, which is supported by the finding of the BafA1-induced caspase-independent cell death could be due to the upregulation of PUMA [62]. These data were further validated by in vivo study through immunohistochemical staining of ULK1, p-FUNDC1 S17, FUNDC1, LC3B, and Bcl-2 in the mice bearing Lon-overexpressed OEC-M1 tumors in the presence or absence of CGP37157 (Fig. 8G).

## Discussion

This study demonstrates that mitochondrial Lon, a matrix-resident stress protein, is an EMC protein that accumulates at EMC sites upon hypoxia, allowing for its interaction with ULK1 kinase complex and phosphorylation of downstream FUNDC1-S17. Phosphorylation of FUNDC1-S17 at the EMC sites by ULK1 is necessary for the FUNDC1 binding to LC3 that triggers mitophagy (Fig. 8H). Our findings explore the role of mitochondrial Lon in mitophagy in response to hypoxia or ROS, which facilitates hypoxia-induced drug resistance in cancer therapy.

The maintenance of mitochondrial quality depends on the mitophagy upon stress condition that is shared with several signaling components in every step of autophagic process. The intriguing issue is to identify the factors responsible for triggering the mitophagy from the matrix inside of mitochondria in response to hypoxia. On the other hand, although mitochondrial Lon has been identified as an MAM (ER-mitochondria contact sites, EMC) protein under stress [33], the real physiological function of mitochondrial Lon remains still unclear. In the present study, our findings extend the recent study to establish a chaperone role of mitochondrial Lon in regulating autophagosome formation at the EMC site during mitophagy. We provide the evidence that mitochondrial matrix protease Lon interacts with ULK1 kinase complex at EMC sites to drive mitophagy upon hypoxia. The ULK1 kinase, one of the upstream components of autophagy, is important for recruitment and phosphorylation of its complex partners, ATG13/FIP200/ATG101. This complex was highlighted for the stability and kinase activity of ULK1 for the functional macroautophagy/selective autophagy [13]. The complex stability is affected when ULK1 is repressed by certain energy sensors like mTOR during nutrient-replete status [19, 63]. On the other hand, p32/C1QBP was reported to stabilize the ULK1 to promote its kinase activity for macroautophagy under starvation [48]. However, how the ULK1 kinase stability is improved to overcome repression to adapt the mitophagy under hypoxia is still not fully clear. In agreement with this scenario, the present study extends the recent study [33] to aim at a chaperone role of mitochondrial Lon in regulating the ULK1 complex at autophagosome formation for mitophagy during hypoxia. Through this study we have provided the evidence about the translocation of mitochondrial Lon to EMC and further promoting the interaction with the ULK1 complex, which is imperative to how signaling from the inside mitochondria to trigger the mitophagy involved in clearance of damaged mitochondria during hypoxic stress.

During hypoxia, Lon translocates from mitochondria to EMC to interact with FUNDC1-ULK1 and its complex partners and allow them enriched in the EMC, which further activates ULK1 activity to phosphorylate FUNDC1 at serine 17. This promotes the FUNDC1 to recognize and bind with LC3 that promotes the mitophagosome fusion with lysosomes. FUNDC1 acts as a key hypoxia-induced mitophagy receptor and is tightly regulated by posttranslational modification. At the early stage of hypoxia, FUNDC1-mediated mitophagy activity is inhibited by its phosphorylation by Src/CK2 kinase and ubiquitination-mediated degradation by MARCH5 [64], thus protecting mitochondria from unnecessary degradation. As hypoxia progress, the interaction of FUNDC1 with Src/CK2 gradually decreases and FUNDC1 begins accumulating at ER-mitochondria contact sites, allowing an abundance of FUNDC1 at the MAM stabilized by USP19 for hypoxia-induced mitochondrial division [65]. We also found that the phosphorylation of Drp1 at serine 616 for mitochondrial fission was increased first and then decreased whereas fusion (p-DRP1 serine 637) was increased later (Fig. S4C). Our results were consistent with the recent report on DRP1-mediated mitochondrial fission showing “eat me signal” by marking the damaged mitochondria under hypoxia [66].

Molecular chaperone Lon plays important roles in promoting cell survival and tumor growth under oxidative and hypoxic stress [22–24, 31]. We revealed, for the first time, the mechanism of how mitochondrial Lon regulates mitophagy and cell survival in the EMC. A growing list of proteins have been identified as the EMC constituents, but how Lon is recruited and functions during stress situations is still not known. The mechanisms involved in the EMC assembly are still not fully understood, which limits our knowledge of how signal transduction from mitochondria triggers the interaction between the two organelles. Most importantly, Lon is involved in the inter-organellar crosstalk between the ER and mitochondria as a EMC protein itself. The scenarios for the mechanism underlying Lon enhanced cell survival and mitophagy through binding with ULK1 complex are proposed. ULK1 kinase stability or its phosphorylation status determines its involvement in autophagy regulation or mitophagy towards cancer progression [67]; ULK1 was identified to be associated with the poor prognosis of patients with lung cancer [68]. Our data showed that ULK1 is phosphorylated and activated by AMPK at Serine 555 in response to hypoxia, which are similar to previous reports [69, 70]. The stability of ULK1 kinase upon CoCl_2_ treatment and Lon upregulation and its downstream activation of mitophagy was strictly increased. However, the treatment of proteasome inhibitor MG132 and NAC blocked the stabilization of ULK1 by Lon, indicating that Lon prevents ULK1 from proteolytic destruction and Lon- induced ROS might have a crosstalk role in the ubiquitination/deubiquitylation process of ULK1. Through our data, we brought a new dimension in the regulation of ULK1 signaling maintenance by chaperone Lon upregulation upon mitophagy demand. Chaperone Lon is evolving to be recognized for its cyto-protective role in cancer, which is supported by the observation that ULK1 association with the ATPase domain of Lon is important for its maintenance and kinase activity for mitophagy and cell survival under hypoxia. In the future, the mechanism of how mitochondrial Lon facilitates the demand changes in increased mitochondrial ROS, altered Ca^2+^ flux, or altered lipid biosynthesis for signaling to induce morphological changes at the EMC sites is worth further investigating upon the physiological stress demands during cancer therapy.

In conclusion, our findings reveal that under hypoxia, the chaperone property of mitochondrial Lon integrates mitochondrial quality maintenance and mitophagy at the interface of the EMC for cell survival of cancer through the Lon-ROS-ULK1-FUNDC1 axis. Targeting the chaperone activity of Lon and EMC function may be the new strategy in cancer therapy.

## Supplementary information

Supplementary material

checklist

## Data Availability

All data needed to evaluate the conclusions of the paper are present in the main text and supplemental material. Data sharing not applicable as no datasets generated and/or analyzed for this study. Additional data related to this paper may be requested from the corresponding author.
