# Correction: Mitochondrial Lon-induced mitophagy benefits hypoxic resistance via Ca^2+^-dependent FUNDC1 phosphorylation at the ER-mitochondria interface

**DOI:** 10.1038/s41419-025-08273-w

**Published:** 2025-12-23

**Authors:** Ananth Ponneri Babuharisankar, Cheng-Liang Kuo, Han-Yu Chou, Vidhya Tangeda, Chi-Chen Fan, Chung-Hsing Chen, Yung-Hsi Kao, Alan Yueh-Luen Lee

**Affiliations:** 1 PhD program in molecular medicine, NHRI & NCU, Taoyuan, Taiwan; 2 National Institute of Cancer Research, National Health Research Institutes, Zhunan, Miaoli, 35053 Taiwan; 3 Department of Life Sciences, College of Health Sciences & Technology, National Central University, Zhongli, Taoyuan, 32001 Taiwan; 4 Department of Medical Laboratory Science and Biotechnology, Yuanpei University of Medical Technology, Hsinchu, 300 Taiwan; 5 Graduate Institute of Biomedical Sciences, China Medical University, Taichung, 40402 Taiwan; 6 Department of Biotechnology, College of Life Science, Kaohsiung Medical University, Kaohsiung, 80708 Taiwan

**Keywords:** Mitochondria, Mitophagy

Correction to: *Cell Death and Disease* 10.1038/s41419-023-05723-1, published online 16 March 2023

In this article, the authors noticed an error in the WB blot of Figure 7F in the results section. The authors found that the blot of p-FUNDC1-S17 is misplanted and would like to exchange and clarify the experimental blot of p-FUNDC1-S17 in the left panel of Figure 7F. It should be noted that the correction does not change the results and description in the article.

**Original Figure 7** 
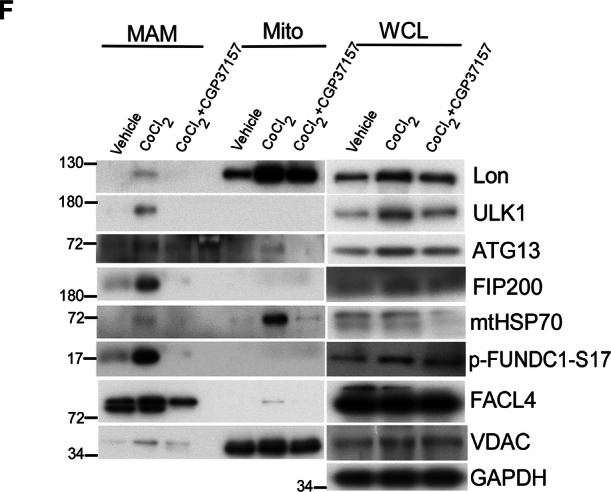


**Amended Figure 7** 
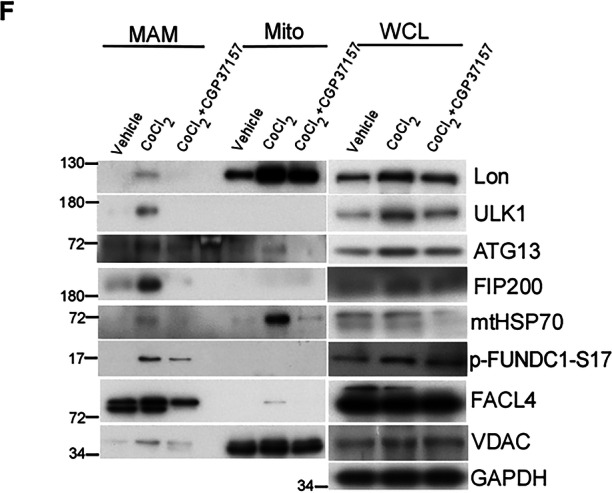


The original article has been corrected.

