# Supplementary material for: Mitochondrial Lon-induced mitophagy benefits hypoxic resistance via Ca2+-dependent FUNDC1 phosphorylation at the ER-mitochondria interface
Source: Cell Death Dis. 2023 Mar 16;14(3):199. doi: 10.1038/s41419-023-05723-1 (PMC10020552; doi:10.1038/s41419-023-05723-1)
Supplement: Supplementary file 1 — Supplementary material [file 41419_2023_5723_MOESM1_ESM.docx]

**Supplementary material**

**Mitochondrial Lon-induced mitophagy benefits hypoxic resistance via Ca^2+^-dependent FUNDC1 phosphorylation at the ER-mitochondria interface**

Ananth Ponneri Babuharisankar ^1,2,3^, Cheng-Liang Kuo^2^, Han-Yu Chou^2^, Vidya Tangeda^1,2,3^, Chi-Chen Fan^4,5^, Chung-Hsing Chen^1^, Yung-Hsi Kao^1,3^, and Alan Yueh-Luen Lee^1,2,3,6,7^*

*: Corresponding author:

National Institute of Cancer Research, National Health Research Institutes, 35 Keyan Road, Zhunan, Miaoli 35053, Taiwan

Tel.: +886-37-206-166 ext. 31705

Fax: +886-37-586-463

E-mail: alanylee@nhri.edu.tw

ORCID ID: 0000-0003-0252-0571


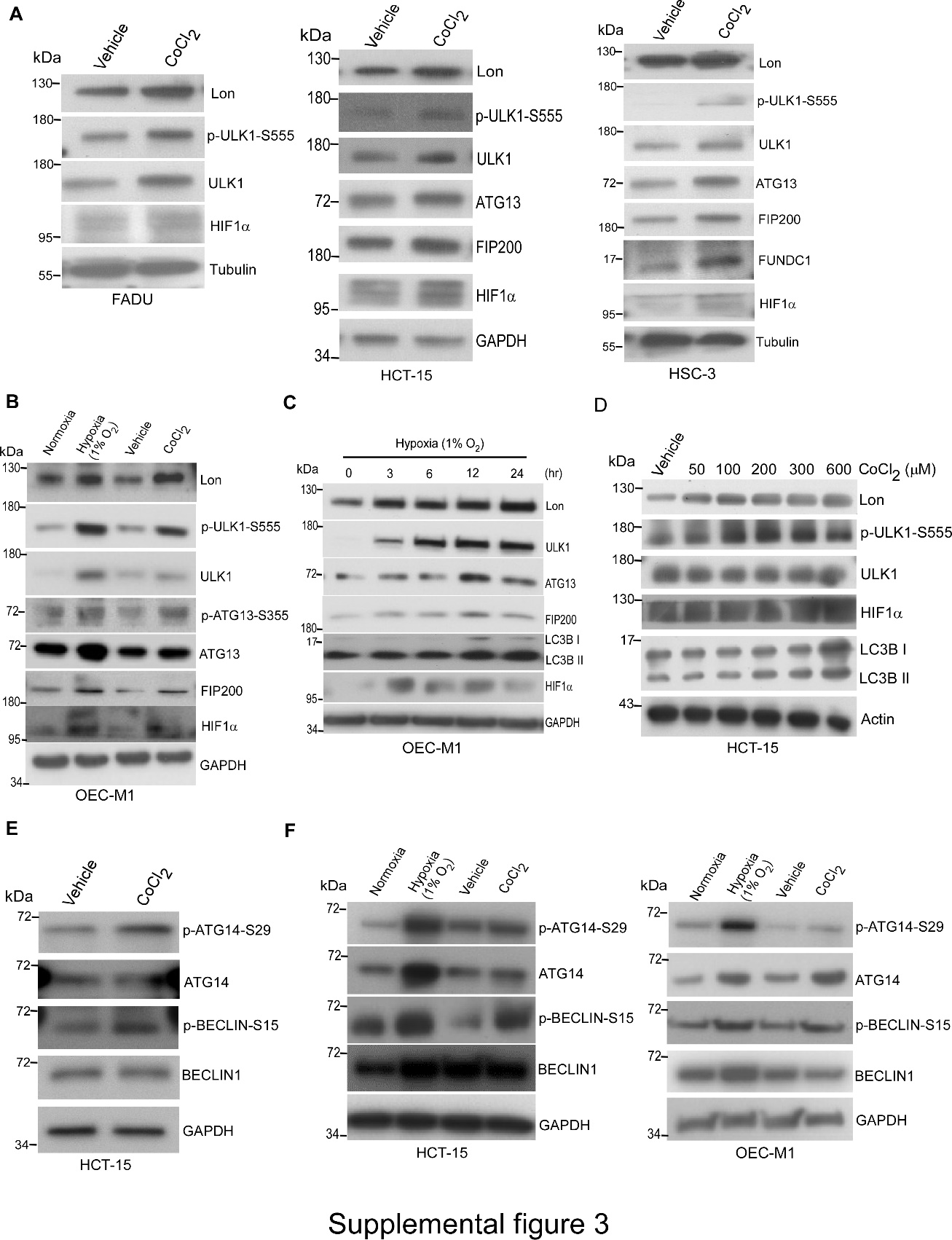


**Figure S1 Hypoxia induces mitophagy by upregulating Lon and ULK1 signaling**

1. FADU, HCT-15, and HSC3cells were exposed to CoCl_2_ (200 µM) for 16 h and the collected lysates were immunoblotted against the mitophagy signaling using indicated antibodies. GAPDH and Tubulin as the loading control.
2. OEC-M1 cells were exposed to hypoxia (1% O_2_) for 24 h or CoCl_2_ (200 µM) for 16 h and the collected lysates were immunoblotted against the mitophagy signaling using indicated antibodies. GAPDH as the loading control.
3. OEC-M1 cells were exposed to hypoxia (1% O_2_) for 24 h for 16 h and the collected lysates were immunoblotted against the mitophagy signaling using indicated antibodies. GAPDH as the loading control.
4. HCT-15 cells were treated with CoCl_2_ for different concentration and the indicated protein expression levels were determined after western blotting. Actin as the loading control.
5. HCT-15 cells were exposed to CoCl_2_ (200 µM) for 16 h and the collected lysates were immunoblotted using indicated antibodies. GAPDH as the loading control.
6. HCT-15 and OEC-M1 cells were exposed to hypoxia (1% O_2_) for 24 h or CoCl_2_ (200 µM) for 16 h and the collected lysates were immunoblotted against the mitophagy signaling using indicated antibodies. GAPDH as the loading control.

**
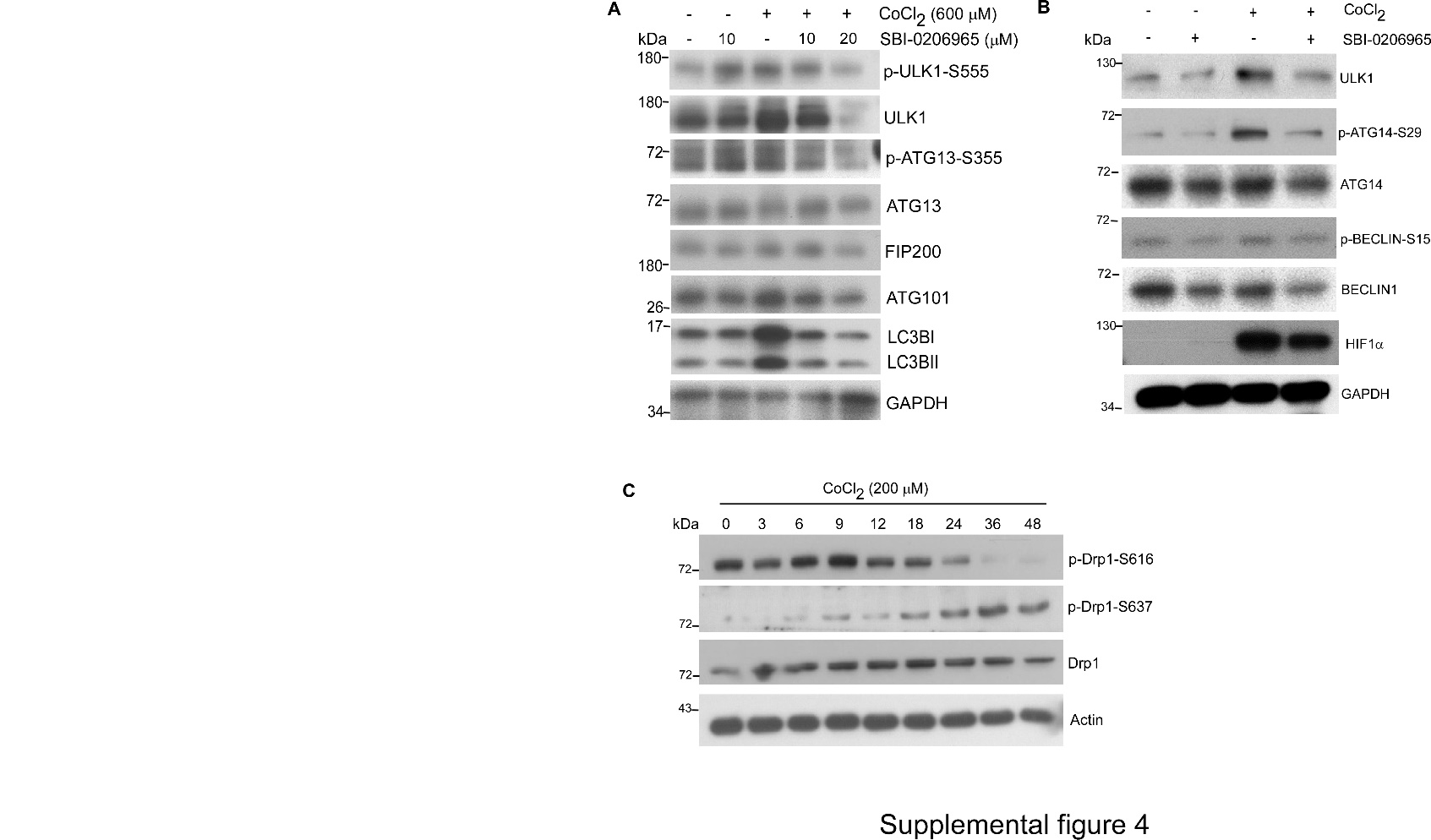
**

**Figure S2 CoCl_2_ induces mitophagy by upregulating Lon and ULK1 signaling**

1. HCT-15 cells were treated with CoCl_2_ (200 μM for 18 h) or not in the presence or absence of SBI-0206965 (20 μM for 6 h). Cell lysates were analyzed by immunoblotting using the indicated antibodies. GAPDH as the loading control.
2. HCT-15 cells were treated with CoCl_2_ (200 μM for 18 h) or not in the presence or absence of SBI-0206965 (20 μM for 6 h). Cell lysates were analyzed by immunoblotting using the indicated antibodies. GAPDH as the loading control.
3. HCT-15 cells were treated with 200 μM/mL CoCl_2_ for different time points (0-48h) and the indicated protein expression levels were determined after western blotting.

**
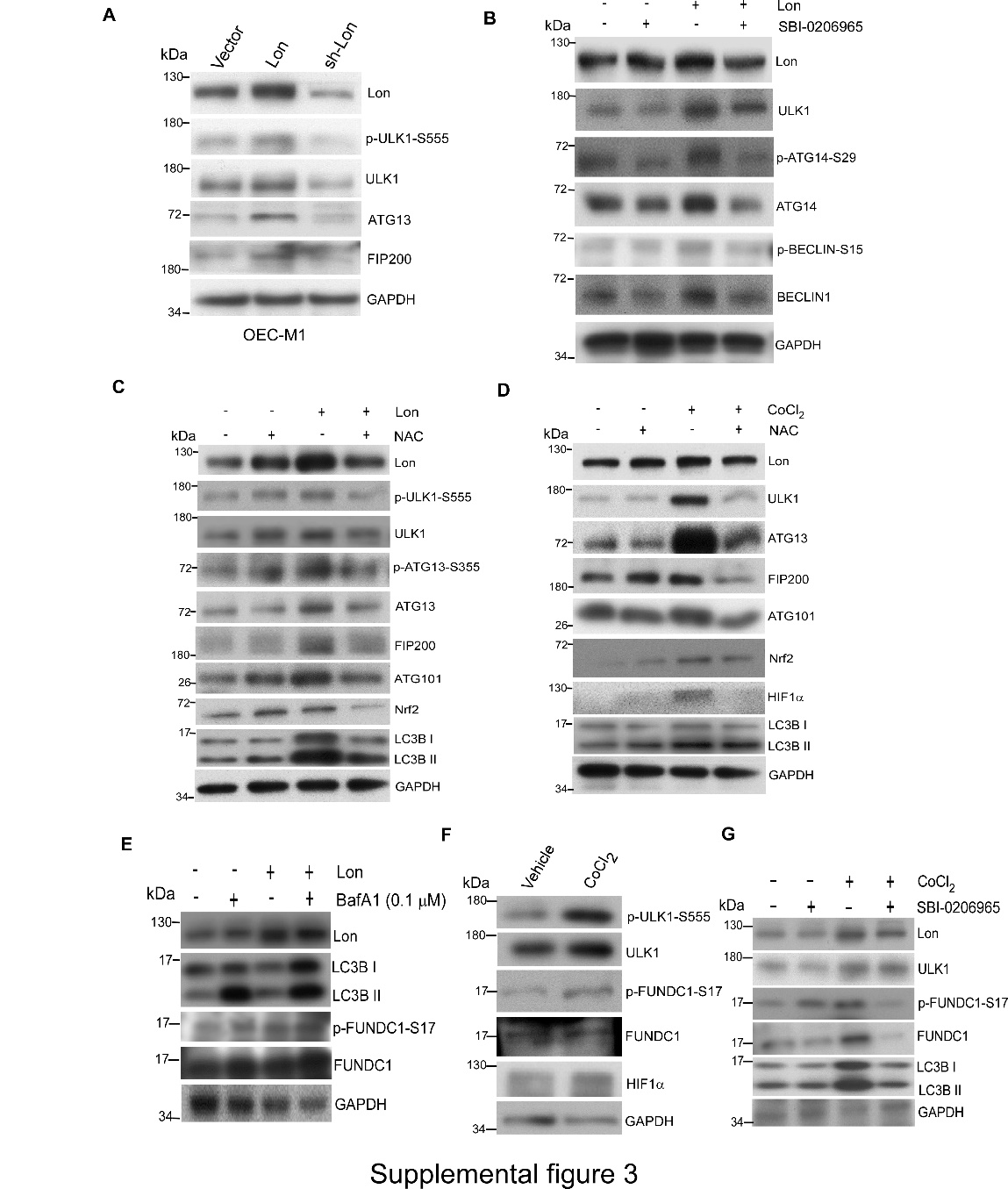
**

**Figure S3 Lon induced ROS contributes for ULK1 signaling activation and mitophagy**

1. OEC-M1 cells were transiently transfected with the plasmids encoding Lon or Lon-shRNA. Western blotting was performed using the indicated antibodies. GAPDH as the loading control.
2. HCT-15 cells were transiently transfected with the plasmids encoding Lon or empty vector in the presence or absence of SBI-0206965 (20 µM for 6h). Western blotting was performed using the indicated antibodies. GAPDH as the loading control.
3. & (D) HCT-15 cells were transiently transfected with the plasmids encoding Lon or empty vector or treated with CoCl_2_ (200 µM) in the presence or absence of NAC (300 uM for 24h). Western blotting was performed to determine the ULK1 complex signaling using the indicated antibodies. GAPDH as the loading control.
4. HCT-15 cells transiently expressing Lon in the presence or absence of BafilomycinA1 (100 nM for 6h). Western blotting was performed using the indicated antibodies. GAPDH as the loading control.
5. HCT-15 cells were exposed to CoCl_2_ (200 µM) for 16 h and the collected lysates were immunoblotted against the mitophagy signaling using indicated antibodies. GAPDH as the loading control.
6. HCT-15 cells were exposed to CoCl_2_ (200 µM) for 16 h in the presence or absence of SBI-0206965 (20 µM for 6h). Western blotting was performed using the indicated antibodies. GAPDH as the loading control.


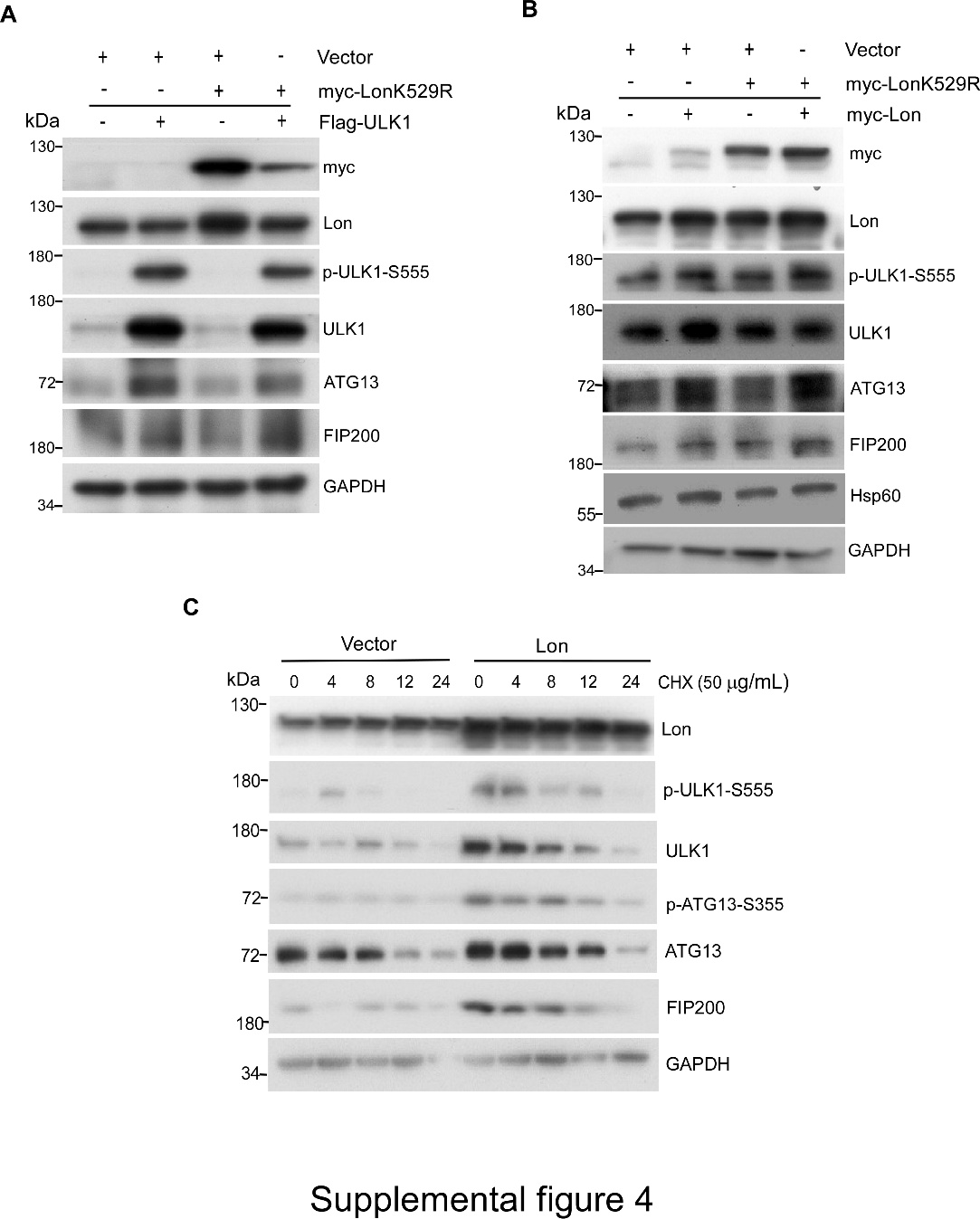


**Figure S4 Lon chaperone mutation significantly inhibits ULK1 signaling activation**

1. HCT-15 cells were transiently transfected with the plasmids encoding myc-LonK529R both individually and together with Flag-ULK1. Western blotting was performed using the indicated antibodies.
2. HCT-15 cells were transiently transfected with the plasmids encoding myc-LonK529R both individually and together with myc-Lon. Western blotting was performed using the indicated antibodies. Hsp60 was used a positive control.
3. HCT-15 cells transfected with the plasmids encoding Lon or empty vector were treated with or without Cycloheximide (50 µg/mL) for the indicated time course . Cell lysates were analyzed by immunoblotting using the indicated antibodies. GAPDH as the loading control.

**
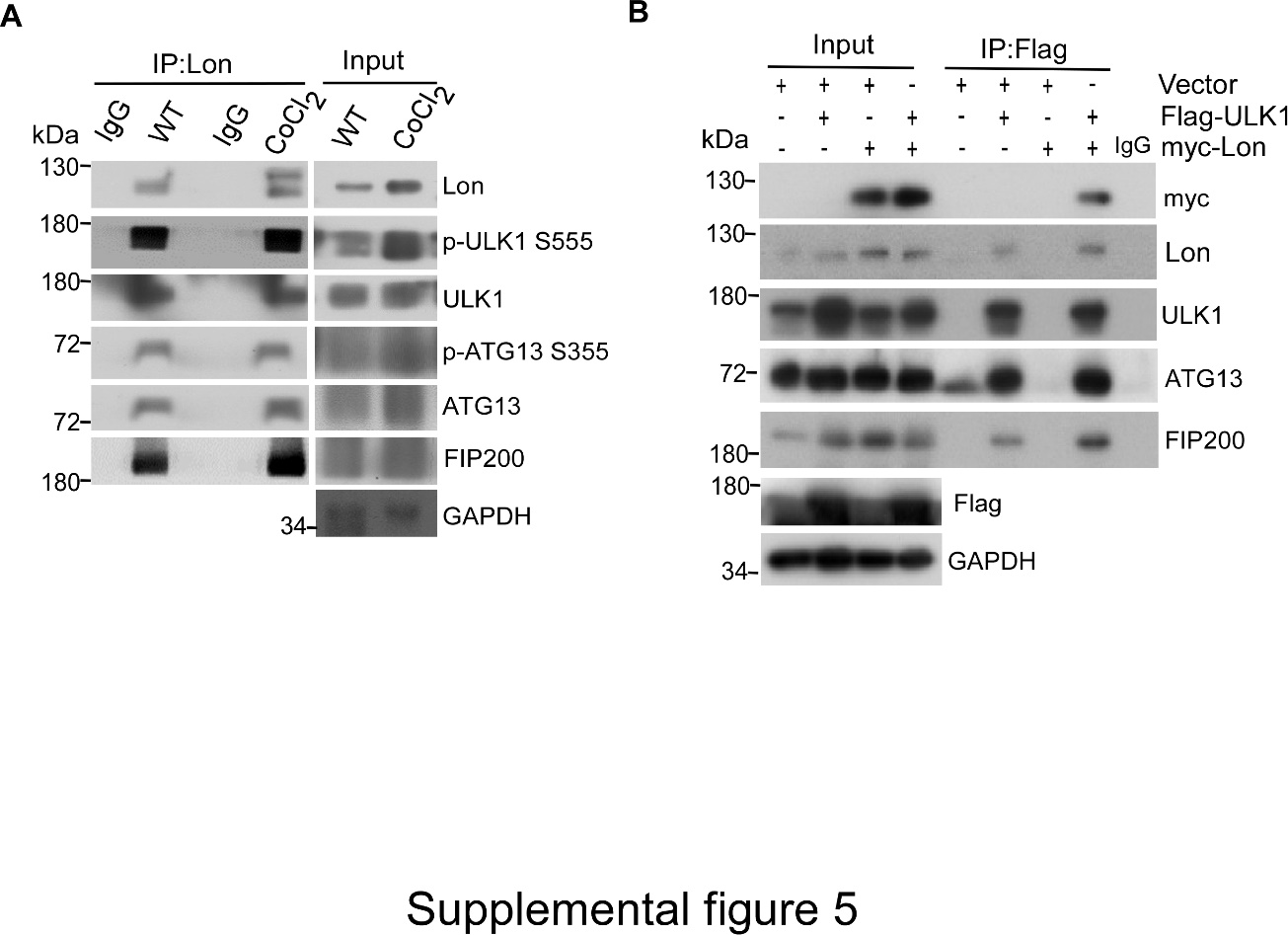
**

**Figure S5 Lon interacts with ULK1 complex**

1. Western analysis of immunoprecipitation assay of endogenous Lon in CoCl2 (200 μM/mL-18h) treated HCT-15 cells.
2. Western analysis of immunoprecipitation assay for Flag in wild type (WT), myc-Lon and Flag-ULK1 expressed HCT-15 cells.


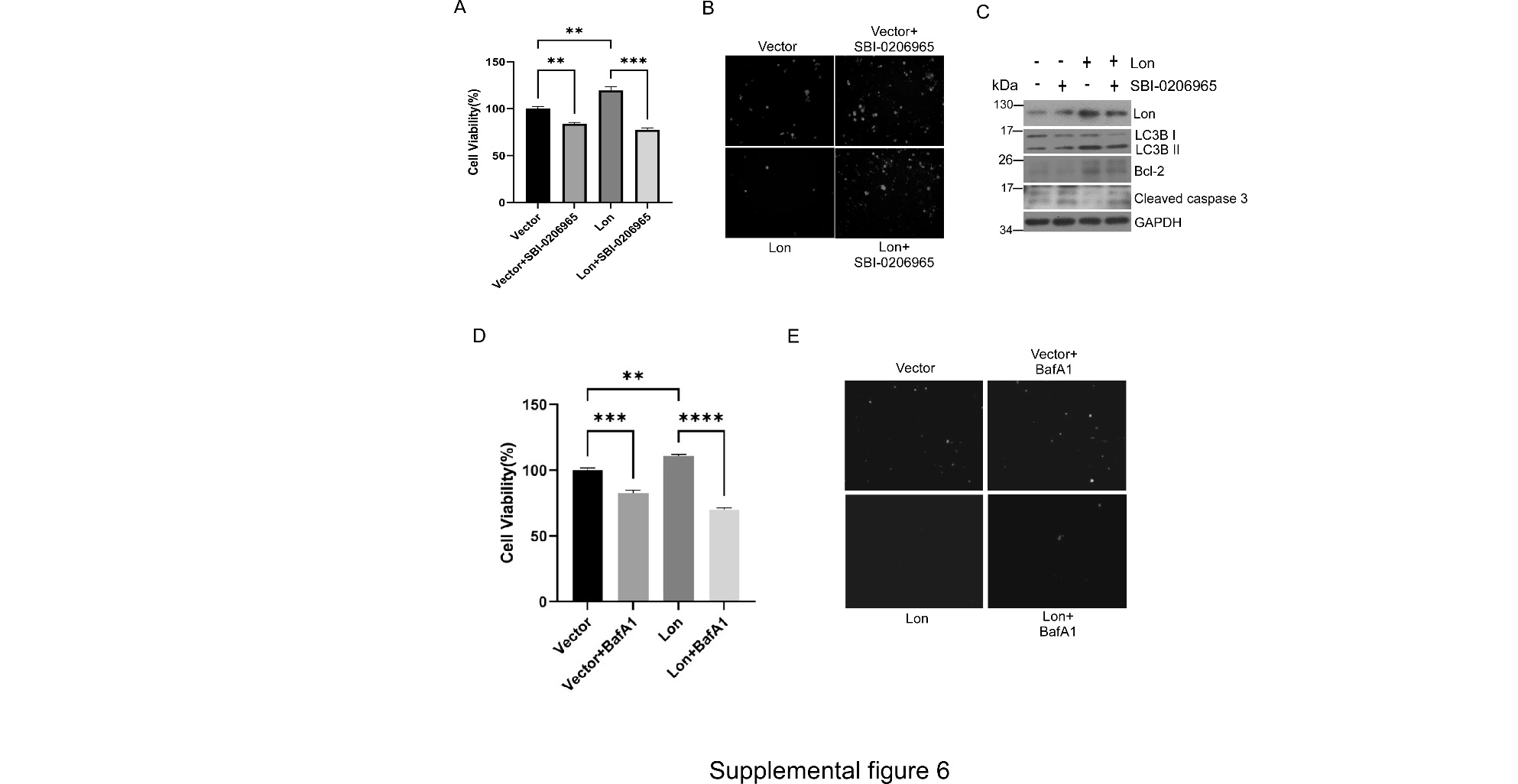


**Figure S6 Bafilomycin A1 significantly inhibits Lon induced mitophagy and cell viability**

(A-C) HCT-15 cells were transfected with the plasmids encoding Lon or empty in the presence or absence of SBI-0206965 (20 μM for 24 h). The MTS assay for cell viability (D), fluorescence-based cleaved Caspase 3 apoptosis assay (E), and Western blotting analysis (F) were performed. Immunoblots were obtained using the indicated antibodies. Scale bar, 100 μm (n= >50 cells/condition and 3 biological replicates).

(D) MTS assay performed in Lon transiently expressed HCT-15 cells in presence or absence of Bafilomycin A1 (100nM for 24h).

(E) Fluorescence based Cleaved Caspase 3 dependent apoptosis assay were performed in Lon transiently expressed HCT-15 cells in presence or absence of Bafilomycin A1 (100nM for 24h). Add 1uL of Nucview® 488 dye (200X dilution) in the medium to treat the cells and incubate for 30 minutes at 37°C. Analyze fluorescence by fluorescence microscopy using 488nm excitation.
